# GATA4-driven miR-206-3p signatures control orofacial bone development by regulating osteogenic and osteoclastic activity

**DOI:** 10.7150/thno.58052

**Published:** 2021-07-25

**Authors:** Shuyu Guo, Jiawen Gu, Junqing Ma, Rongyao Xu, Qingheng Wu, Li Meng, Haojie Liu, Lu Li, Yan Xu

**Affiliations:** 1 Jiangsu Key Laboratory of Oral Diseases, Nanjing Medical University, 140 Hanzhong Road, Nanjing 210029, China.; 2 Department of Periodontology, The Affiliated Stomatological Hospital of Nanjing Medical University, Nanjing, China.; 3 Department of Orthodontics, The Affiliated Stomatological Hospital of Nanjing Medical University, Nanjing, China.; 4 Jiangsu Province Engineering Research Center of Stomatological Translational Medicine, Nanjing, China.

## Abstract

Growth disorders in the orofacial bone development process may lead to orofacial deformities. The balance between bone matrix formation by mesenchymal lineage osteoblasts and bone resorption by osteoclasts is vital for orofacial bone development. Although the mechanisms of orofacial mesenchymal stem cells (OMSCs) in orofacial bone development have been studied intensively, the communication between OMSCs and osteoclasts remains largely unclear.

**Methods:** We used a neural crest cell-specific knockout mouse model to investigate orofacial bone development in GATA-binding protein 4 (GATA4) morphants. We investigated the underlying mechanisms of OMSCs-derived exosomes (OMExos) on osteoclastogenesis and bone resorption activity *in vitro*. miRNAs were extracted from OMExos, and differences in miRNA abundances were determined using an Affymetrix miRNA array. Luciferase reporter assays were used to validate the binding between GATA4 and miR-206-3p in OMSCs and to confirm the putative binding of miR-206-3p and its target genes in OMSCs and osteoclasts. The regulatory mechanism of the GATA4-miR-206-3p axis in OMSC osteogenic differentiation and osteoclastogenesis was examined *in vitro* and *in vivo*.

**Results:** *Wnt1-Cre;Gata4^fl/fl^* mice (cKO) not only presented inhibited bone formation but also showed active bone resorption. Osteoclasts cocultured *in vitro* with cKO OMSCs presented an increased capacity for osteoclastogenesis, which was exosome-dependent. Affymetrix miRNA array analysis showed that miR-206-3p was downregulated in exosomes from shGATA4 OMSCs. Moreover, the transcriptional activity of miR-206-3p was directly regulated by GATA4 in OMSCs. We further demonstrated that miR-206-3p played a key role in the regulation of orofacial bone development by directly targeting bone morphogenetic protein-3 (Bmp3) and nuclear factor of activated T -cells, cytoplasmic 1 (NFATc1). OMExos and agomiR-206-3p enhanced bone mass in *Wnt1-cre;Gata4^fl/fl^*mice by augmenting trabecular bone structure and decreasing osteoclast numbers.

**Conclusion:** Our findings confirm that miR-206-3p is an important downstream factor of GATA4 that regulates the functions of OMSCs and osteoclasts. These results demonstrate the efficiency of OMExos and microRNA agomirs in promoting bone regeneration, which provide an ideal therapeutic tool for orofacial bone deformities in the future.

## Introduction

Three-quarters of human birth defects are related to craniofacial malformations (http://www.cdc.gov/ncbddd/bd/centers.htm, Centers for Disease Control, Birth Defects Research and Prevention). Orofacial mesenchymal stem cells (OMSCs), which are derived from neural crest cells (NCCs) and differentiate into osteoblasts, are the main cells participating in the development of orofacial bone 1-3. Although orofacial bone is formed by OMSC-derived osteoblasts, there are also large numbers of mesoderm-derived cells, such as osteoclasts, on the bone surface 4, 5. This elaborate arrangement may be important for orofacial bone development because studies have suggested that an imbalance in the interactions between mesenchymal stem cells (MSCs) and osteoclasts can have profound effects on bone homeostasis 6-8.

Previous studies on the development of orofacial bone generally focused on the regulatory mechanism of MSCs participating in bone osteogenic differentiation 9, 10. However, the communication between OMSCs and osteoclasts in orofacial bone development has not been studied in depth in recent years. Studies have revealed that exosomes, key mediators that are 40 - 100 nm in diameter, are involved in signal transduction and intercellular substance exchange 11-14. Exosomes can directly transfer genetic material and proteins, such as miRNAs, mRNAs, and cell membrane receptors, to target cells, and exogenous exosomal molecules can be transferred from cell to cell, resulting in cross-cellular gene regulation. Because few studies have examined how OMSC-derived exosomes influence the orofacial bone microenvironment, understanding the regulatory mechanisms of OMSC-derived exosomes in orofacial bone development may be significant for the treatment of orofacial malformations.

GATA-binding protein 4 (GATA4) is a zinc-finger transcription factor, that plays vital roles in bone development. It is well documented that GATA4 transcripts are found within migratory NCCs, and also widely expressed in various neural crest derivatives, including mandible, teeth, and palate 15, 16. GATA4 regulates runt-related transcription factor 2 (Runx2), transforming growth factor beta (TGFβ), and other key proteins in osteoblast function and differentiation 17, 18. Global knockout of *GATA4* in mouse embryos leads to early lethality 19. Mice with conditional knockout of *GATA4* in osteoblasts exhibit reduced bone mineral density 17, 18. Further, mouse NCCs lacking GATA4 show developmental defects in orofacial bone 16, suggesting that GATA4 has an important role in orofacial bone development.

In the present study, we tried to explain why the *Wnt1-Cre;Gata4^fl/fl^* mice display an increased osteoclast differentiation in addition to a reduced osteoblast differentiation. We found that OMSC-derived exosomal miR-206-3p could transfer to osteoclasts to inhibit osteoclastic bone resorption. Moreover, we evaluated the effect of GATA4-driven miR-206-3p signature to elucidate the underlying mechanisms in osteoblastic differentiation, and focused on the potential therapeutic effects of OMSC-derived exosomes on osteoclast activity *in vitro* and conditional knockout of *GATA4* in NCCs-induced osteoporosis mouse model *in vivo*. This study provides a promising therapeutic intervention in orofacial bone development.

## Results

### *Wnt1-cre;Gata4^fl/fl^* mice present retreated mandibles due to decreased bone formation and increased bone resorption

**Figure 1:**
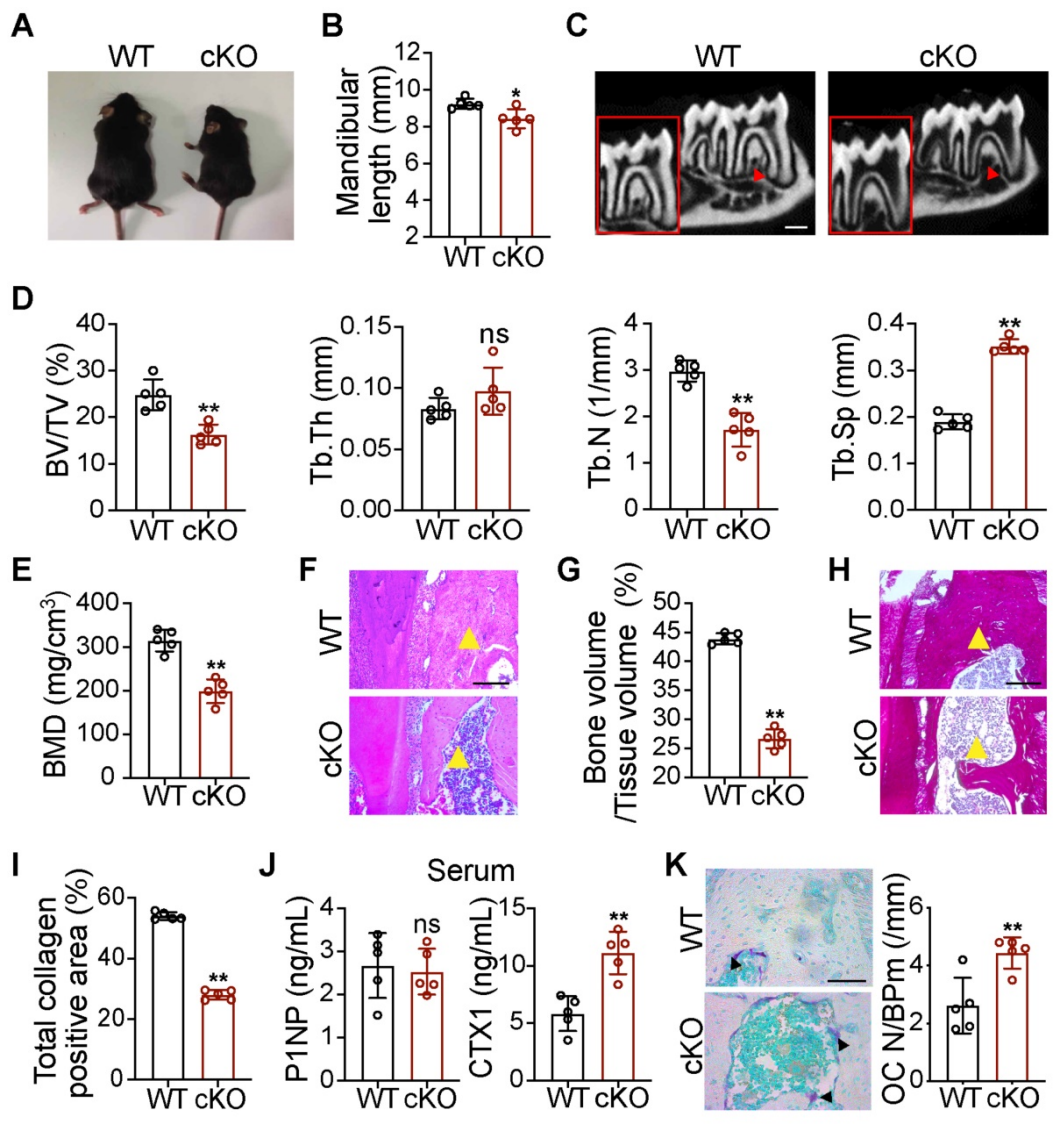
***Wnt1-cre;Gata4^fl/fl^* mice present orofacial bone hypoplasia owing to decreased bone formation and increased bone resorption.** (A) Photographic analysis of *Gata4^fl/fl^* (WT) mice and *Wnt1-cre;Gata4^fl/fl^* (cKO) mice (n = 5). (B) Quantitative analysis of mandibular length in the WT and cKO mice (n = 5). (C) Micro-CT analysis of mandibles from the WT mice and cKO mice at 56 d (n = 5). Decreased mineralization area was denoted by red arrows. Scale bars: 500 µm. (D) Quantification from C. BV/TV, bone volume/tissue volume; Tb.Th, trabecular thickness; Tb.N, trabecular number; Tb.Sp, trabecular separation (n = 5). (E) Bone mineral density (BMD) of the mandibles in the WT and cKO mice (n = 5). (F) Images of H&E staining of mandibles from WT and cKO mice (n = 5). Scale bar: 200 µm. (G) Quantitative analysis of the bone mass, respectively (n = 5). (H) Images of total collagen staining of mandibles from WT and cKO mice (n = 5). Scale bar: 200 µm. Yellow arrows in F and H indicate that cKO mice have a reduced bone mass in the mandible. (I) Quantitative analysis of the positive area of total collagen (n = 5). (J) N-terminal propeptide of type 1 procollagen (P1NP) and carboxy-terminal collagen cross-links (CTX1) were assessed in the serum of 8-week-old male WT and cKO littermates using commercially available ELISAs (n = 5). (K) TRAP staining of WT and cKO mouse mandibles. Osteoclasts were denoted by black arrows (n = 5). OC N/B.Pm (/mm), osteoclast number per bone perimeter. Scale bar: 100 µm. Two-tailed Student's t test. Each experiment was repeated at least three times with the same conditions. Data are shown as mean ± SD. ^*^P < 0.05, ^**^P < 0.01; ns, not significant.

To study the effect of *GATA4* deletion on orofacial bone development, we generated *Wnt1-cre;Gata4^fl/fl^* (cKO) mice, in which *GATA4* was conditionally knocked out in NCCs. The cKO mice were smaller than the wild-type (WT) littermates **(Figure 1A)**. On the basis of the results, cKO mice exhibited mandibular hypoplasia **(Figure 1B)**. As shown in micro-CT images of the mandible, cKO mice had obviously decreased cancellous bone mass **(Figure 1C-E)**. H&E analysis and total collagen staining further indicated a significant reduction in bone mass of the mandible in 56-day-old cKO mice **(Figure 1F-I)**. We then analyzed bone turnover markers in the serum of WT and cKO mice. Serum N-terminal propeptide of type 1 procollagen (P1NP), a marker of bone formation, remained unaltered in cKO mice. Serum carboxyterminal telopeptides of type I collagen (CTX1), a marker of *in vivo* bone-resorption, was significantly increased **(Figure 1J)**. To further explore whether *GATA4* deletion influences the differentiation of bone-resorption cells, osteoclasts were counted in WT and cKO mice. Osteoclasts are derived from the mesoderm and are not derivatives of NCCs; however, tartrate-resistant acid phosphatase (TRAP) staining suggested that cKO mice had an increased number of osteoclasts in the mandible compared with WT mice **(Figure 1K)**. These results suggest that the decreased bone mass in cKO mice may be due to increased bone turnover compared with WT controls, resulting in a negative balance. Thus, it is of interest whether NCC-derived OMSCs could communicate with osteoclasts in the mandibular bone marrow microenvironment.

### OMSCs promote osteoclastic differentiation and function through exosomes

**Figure 2:**
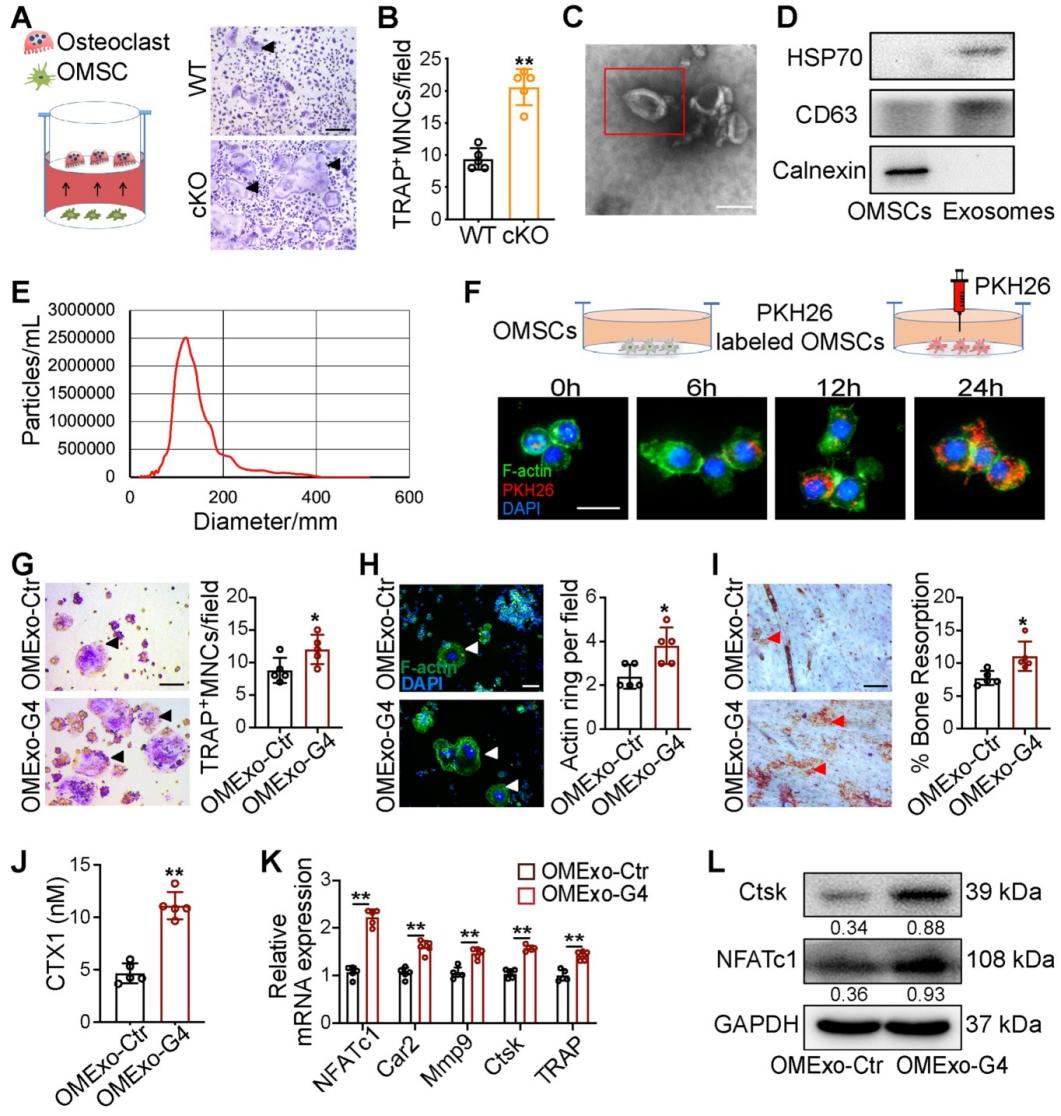
** Exosomes derived from OMSCs promote osteoclastic differentiation and function.** (A) M-CSF and RANKL treated bone marrow-derived macrophages (BMMs) were transfected with supernatant from *Gata4^fl/fl^* (WT) mice and *Wnt1-cre;Gata4^fl/fl^* (cKO) OMSCs, and then the cells were stained for TRAP (n = 5). Scale bar: 200 µm. (B) Quantification of TRAP-positive multinucleated cells (TRAP^+^MNCs). (C) Exosomes were visualized on the TEM. Scale bar: 100 nm. (D) Protein expressions of CD63, HSP70 and Calnexin were detected in OMSCs and exosomes by western blotting. (E) NTA analysis determined the size of exosomes. (F) PKH26-labeled exosomes (red) from OMSCs (OMExos) entering into osteoclasts were observed through confocal microscopy. Fluorescein phalloidin-FITC (green) was used to stain F-actin, while DAPI (blue) was used to stain nuclei. Scale bar, 50 μm. (G) Exosomes isolated from shCtr or shGATA4 OMSCs (OMExo-Ctr, OMExo-G4) conditioning medium were co-cultured with RANKL induced RAW264.7 cells. TRAP staining (black arrows), F-actin ring (white arrows) number per field (H), bone resorption pits (red arrows) using WGA (wheat germ agglutinin) staining (I) were used to detect osteoclast formation and bone resorption (n = 5). Scale bar, 200 μm. (J) Culture medium carboxy-terminal collagen cross-links (CTX1) were assessed by ELISA (n = 5). (K) qRT-PCR analysis of osteoclast marker genes (*NFATc1*, *Car2*, *Mmp9*, *Ctsk* and *Trap*) in osteoclasts treated with OMExo-Ctr or OMExo-G4 (n = 5). (L) Protein levels of NFATc1 and Ctsk were examined by using western blotting. Two-tailed Student's t test. Each experiment was repeated at least three times with the same conditions. Data are shown as mean ± SD. ^*^P < 0.05, ^**^P < 0.01.

To investigate the role of OMSCs in osteoclastogenesis regulation, we co-cultured primary bone marrow-derived macrophages (BMMs) with OMSCs from WT and cKO mice. We performed immunostaining to examine the surface molecular expression of OMSCs. OMSCs were positive for the MSC-markers CD73 and stem cell antigen 1 (Sca-1) (**Figure S1A**). TRAP-positive multinucleated cells (TRAP^+^MNCs), cell size, and the number of nuclei per osteoclast were increased after the cells were treated with supernatant from cKO OMSCs **(Figure 2A, B, and S1B)**. We also investigated the osteoclast differentiation capacity of the BMMs isolated from WT and cKO mice in absence of the supernatant collected from OMSCs. The TRAP^+^MNCs were quantified with no difference noted in either WT or cKO cells (**Figure S1C**). These results indicate that the supernatant from OMSCs is functional for osteoclastogenesis.

Recently, exosomes have been studied as carriers in intercellular communication. To search for a potential paracrine mechanism in OMSC-to-osteoclast communication, we purified microvesicles from OMSCs and observed them by transmission electron microscopy (TEM) to identify exosomes. Under TEM, exosomes were round or oval with a disc-shaped structure **(Figure 2C)**. CD63 and 70 kDa heat shock protein (HSP70) (exosomal markers) were highly expressed in the exosomes, as detected by western blotting, while calnexin (a cytosolic marker) was almost undetectable **(Figure 2D)**. The average size of the exosomes was found to be ~100 nm by nanoparticle tracking analysis **(NTA; Figure 2E)**. Given that exosomes protected miRNAs from RNase-induced degradation and mediate intercellular communication 20, we next verified whether exosomes were transferred from OMSCs to osteoclasts. The exosomes from OMSCs (OMExos) were traced by PKH26 and co-cultured with osteoclasts. Confocal microscopy showed that the osteoclasts exhibited accumulating red fluorescence over time, suggesting that PKH26-labeled OMExos were taken-up by the osteoclasts **(Figure 2F)**.

To analyze the impact of OMExos on osteoclast bone-resorption activity *in vitro*, we induced RAW264.7 cells treated with exosomes from shCtr or shGATA4 OMSCs (OMExo-Ctr or OMExo-G4) to differentiate into osteoclasts. The knockdown efficiencies of shGATA4 in OMSCs were examined by western blotting and qRT-PCR analysis **(Figure S1D, E)**. TRAP staining data indicated that administration of receptor activator of nuclear factor-κΒ ligand (RANKL) strongly induced osteoclast differentiation, and the presence of exosomes from OMExo-G4 markedly increased the formation of TRAP^+^MNCs, cell size, and the number of nuclei per osteoclast** (Figure 2G, Figure S1F)**. Osteoclasts exhibited 2-fold raised actin belt formation in the OMExo-G4 group, an important feature of mature osteoclasts **(Figure 2H)**. OMExo-G4-treated osteoclasts also formed larger area of resorption pits on bone slices **(Figure 2I)**, which was corroborated by the increased release of CTX1 **(Figure 2J)**. These results suggested that OMExo-G4 promoted osteoclast differentiation and fusion and increased bone resorption activity. We further examined the viability and migration activity of RAW264.7 cells in the presence of OMExo-G4 to determine the underlying reason for OMExo-G4-mediated upregulation of osteoclast differentiation and fusion. By counting kit-8 assay (CCK-8) and wound scratch assay, the viability of exosome-treated RAW264.7 cells increased with time and OMExo-G4-treated and control cells exhibited similar scratch closure times, indicating that the viability and migratory ability of the cells was not affected by OMExo-G4 treatment **(Figure S1G, H)**.

In line with these findings, quantitative reverse transcription PCR (qRT-PCR) analysis confirmed that the mRNA levels of *NFATc1*, carbonic anhydrase II (*Car2*), matrix metalloproteinase-9 (*Mmp9*), cathepsin K (*Ctsk*) and *TRAP* were significantly increased in the OMExo-G4 group compared with the control** (Figure 2K)**. At the protein level, NFATc1 (an osteoclast-related transcription factor) and Ctsk (a gene critical for osteoclast function) were increased in the OMExo-G4 group compared with the control** (Figure 2L)**.

Overall, these data indicate that OMSCs may regulate osteoclastogenesis though OMExos.

### OMSC-derived exosomal miR-206-3p is directly regulated by GATA4

**Figure 3:**
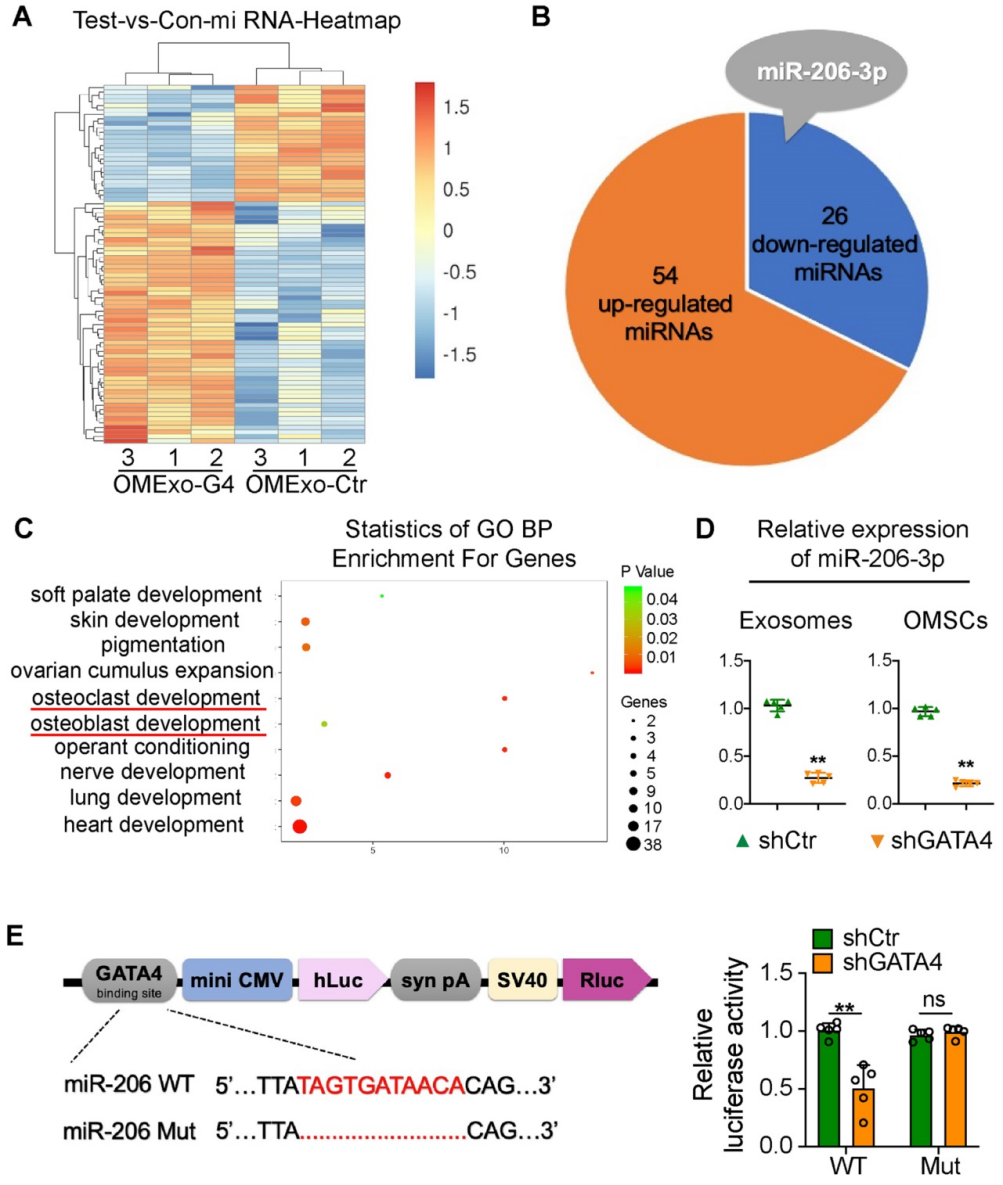
** OMSC-derived exosomal miR-206-3p is directly regulated by GATA4.** (A) Unsupervised hierarchical clustering and heat map diagram of the differential expression miRNAs in exosomes from shCtr or shGATA4 OMSCs (OMExo-Ctr, OMExo-G4) by Affymetrix miRNA 4.0 Array analysis (n = 3). Red color indicates higher expression of miRNAs while blue color indicates lower expression. (B) Fifty-four miRNAs were upregulated and 26 miRNAs were downregulated in OMExos after *GATA4* knockdown. (C) Gene ontology (GO) enrichment bubble plot of target genes of miR-206-3p in biological processes. (D) The relative expression of miR-206-3p in shGATA4 OMSCs and their exosomes were accessed by qRT-PCR (n = 5). (E) JASPAR database was used to forecast several putative GATA4 binding sites upstream of the transcription start site of the mouse miR-206 promoter. The structure diagram of the Dual-Luciferase reporter vector and the sequences of the putative GATA4-binding sites in WT and mutants. Quantitative results of dual-luciferase reporter assay (n = 5). Two-tailed Student's t test. Each experiment was repeated at least three times with the same conditions. Data are shown as mean ± SD. ^**^P < 0.01; ns, not significant.

To identify candidate exosomal miRNAs that potentially participate in the communication between OMSCs and osteoclasts, we performed an Affymetrix miRNA array analysis of OMExos and detected changes in miRNA levels after *GATA4* knockdown. Among the identified miRNAs, 54 miRNAs were significantly upregulated, and 26 miRNAs were significantly downregulated in OMExos after *GATA4* knockdown (**Figure 3A, B; Table S2**). Based on these results and references, we focused on miR-206-3p, which was obviously downregulated in the OMExo-G4 group **(Figure 3B)**. A previous study showed that miR-206-3p has developmental roles in the modulation of Wnt signaling during tooth morphogenesis 21. Additionally, miR-206-3p was down-regulated in ovariectomy (OVX) mice, indicating its potential role in bone metabolism 22. Gene ontology (GO) enrichment analysis indicated that target genes of miR-206-3p are associated with several biological processes, including osteoblast development, osteoclast development, palate development, and neuro development (**Figure 3C**). Following the Affymetrix miRNA array analysis, qRT-PCR analysis showed that the expression of miR-206-3p in shGATA4 OMSCs and their exosomes were decreased** (Figure 3D)**. These results indicate that miR-206-3p might function as a potential mediator of GATA4 in OMSCs and OMExos during orofacial bone development.

It has been reported that GATA4 controls physiological changes in various cells as a transcription factor 23, 24. Therefore, we predicted several putative GATA4-binding sites upstream of the transcription start site of the mouse miR-206 promoter in the JASPAR database, and we chose one predicted binding site with high scores for further research. A dual-luciferase assay was then performed to analyze the promoter region. In the dual-luciferase assay, we constructed several reporter vectors including the GATA4-binding site and a mutant GATA4-binding site. GATA4 inhibition decreased the transcription of miR-206-3p compared with that of the control group, while there was no statistically significant change in miR-206-3p expression in the mutant GATA4-binding site group** (Figure 3E)**. These results indicate that GATA4 directly associates with the miR-206 promoter, which further suggests that GATA4 may be involved in the regulation of bone physiology through miR-206-3p.

### GATA4-miR-206-3p-Bmp3 signaling regulates osteogenic differentiation of OMSCs

To synthetically verify the regulatory mechanism of the GATA4-miR-206-3p axis in the orofacial bone microenvironment, we investigated OMSC osteogenic differentiation. Primary OMSCs were obtained from WT and cKO mice. Compared with the OMSCs from WT group, OMSCs from cKO mice presented decreased alkaline phosphatase activity at day 5 of induction and fewer nodules at day 14 of induction by ARS assay **(Figure S2A)**. In addition, we detected the mRNA levels of several osteogenic genes, including osteopontin (*Opn*), osteocalcin (*Ocn*), *Runx2*, *Alp*, osterix (*Osx*) and *Rankl* in OMSCs transfected with *GATA4* lentivirus. As shown in **Figure S2B**, *GATA4* knockdown in OMSCs increased the expression of *Rankl* and inhibited the expression of the *Opn*, *Ocn*, *Runx2*, *Alp* and *Osx*. At the protein level, the expression of Opn, Ocn, Runx2, Osx also decreased in the shGATA4 group (**Figure S2C**). These results prove the role of GATA4 in the regulation of OMSC osteogenic differentiation.

**Figure 4:**
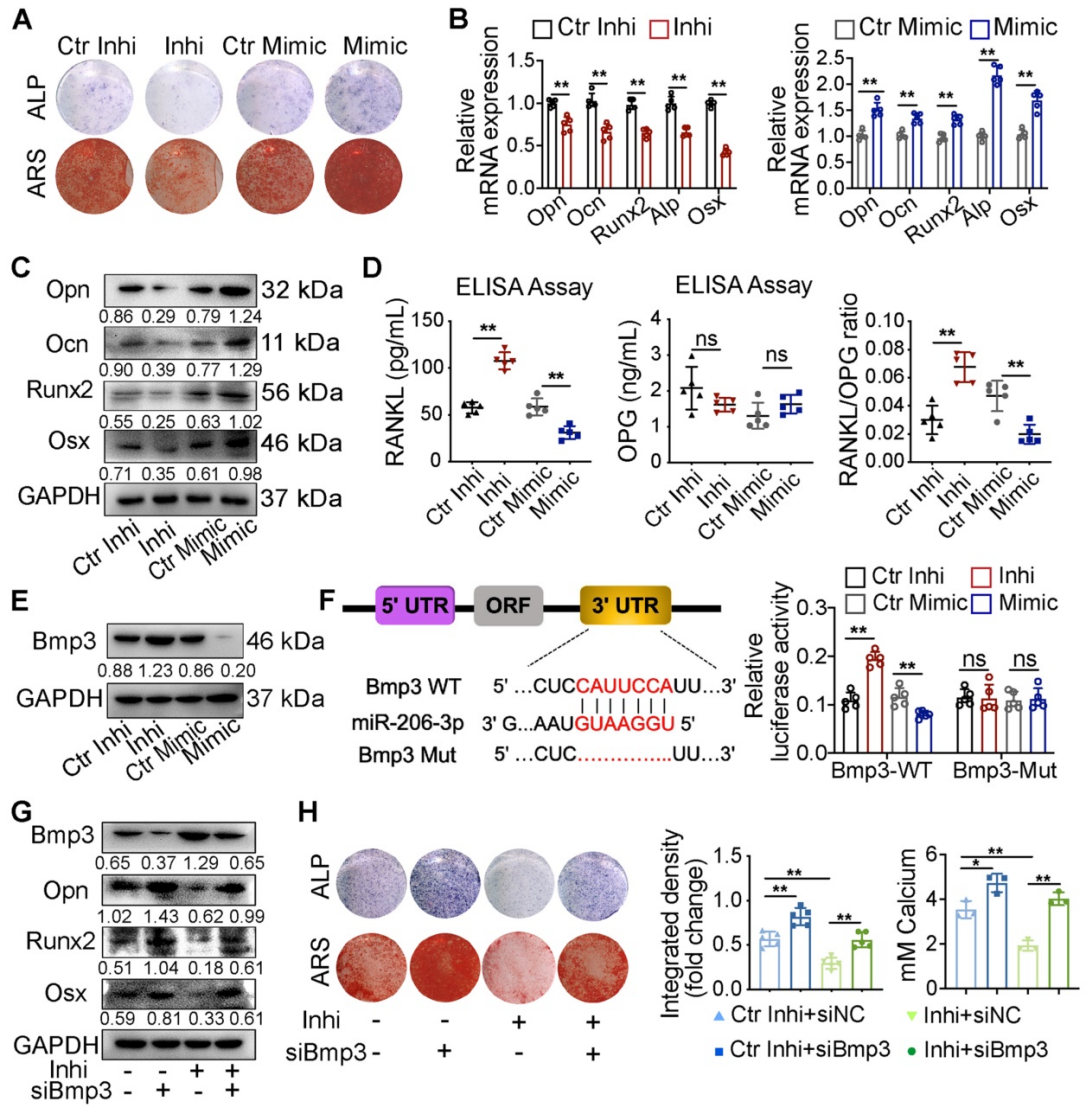
** GATA4-miR-206-3p-Bmp3 signaling in regulating osteogenic differentiation of OMSCs.** (A) The ALP activity and the number of mineral nodules of OMSCs transfected miR-206-3p inhibitor or mimic were accessed by ALP and ARS assay (n = 5). (B) Indicated osteogenic gene expression analysis of miR-206-3p control inhibitor or mimic-transfected OMSCs after 5 d following mineralization induction (n = 5). (C) Protein expression levels of several osteogenic markers in miR-206-3p inhibitor or mimic-transfected OMSCs after 5 d following mineralization induction (n = 5). (D) OMSCs were cultured with miR-206-3p inhibitor or mimic in the osteogenic medium. Three days post-culture, supernatants were obtained to detect the amounts of RANKL, OPG and the ratio of RANKL/OPG in the supernatants by ELISA (n = 5). (E) miR-206-3p inhibitor or mimic transfected OMSCs following mineralization induction were subjected to western blotting analysis to detect the protein levels of Bmp3 (n = 5). (F) The potential binding sites for miR-206-3p on the 3' UTR of Bmp3. Dual-luciferase reporter assays were carried out to verify that miR-206-3p directly targeted the 3' UTR of Bmp3. (G) OMSCs were co-transfected with miR-206-3p inhibitor and siBmp3, the protein levels of Bmp3, Opn, Runx2 and Osx in the indicated cells were examined by using western blotting analysis (n = 5). (H) The ALP activity and the number of mineral nodules of OMSCs co-transfected with miR-206-3p inhibitor and siBmp3 were accessed by ALP (n = 5) and ARS assay (n = 3). control inhibitor, Ctr-inhi; inhibitor, inhi; control mimic, Ctr-mimic. Ordinary one-way ANOVA. Each experiment was repeated at least three times with the same conditions. Data are shown as mean ± SD. ^*^P < 0.05, ^**^P < 0.01; ns, not significant.

As miR-206-3p was directly regulated by GATA4 in OMSCs, we next examined the role of miR-206-3p in the GATA4-mediated promotion of osteogenesis. Following a period of osteogenic induction, the knockdown and overexpression efficiencies of miR-206-3p in OMSCs were evaluated by qRT-PCR **(Figure S2D)**. By ALP and ARS assay, the miR-206-3p inhibitor group showed a decrease in ALP activity and nodules formation, and the miR-206-3p mimic group presented an increase in activity** (Figure 4A, Figure S2E)**. In the miR-206-3p inhibitor group, osteogenesis-related genes exhibited lower levels; however, miR-206-3p overexpression in OMSCs increased the levels of osteogenic markers **(Figure 4B, C)**. As determined by ELISA of the culture media, miR-206-3p knockdown or overexpression did not affect OPG but enhanced the protein level of RANKL and the ratio of RANKL to OPG in miR-206-3p inhibitor group while decreased in miR-206-3p mimic group during the osteogenic differentiation of OMSCs **(Figure 4D)**. We then investigated whether GATA4-mediated osteogenic differentiation requires miR-206-3p. We found that transfection of OMSCs with miR-206-3p mimic reversed the repressive effect of GATA4 shRNA on osteogenesis and osteogenic gene expression **(Figure S2F, G)**.

Bmp3 has an inhibitory role in the bone formation of osteoblasts 25, 26. qRT-PCR and western blotting analyses showed that expression of *Bmp3* was significantly increased in OMSCs treated with miR-206-3p inhibitor and decreased in the miR-206-3p mimic group **(Figure 4E, S2H)**. Additionally, the expression level of Bmp3 was also increased in OMSCs from cKO mice compared with WT mice (**Figure S3I**). Bioinformatics analysis predicted Bmp3 as a potential target gene of miR-206-3p. According to dual-luciferase reporter assays in 293T cells, miR-206-3p directly binds the 3'-untranslated region (3'UTR) of Bmp3 and resulting in translational inhibition **(Figure 4F)**.

To further confirm the role of GATA4-miR-206-3p-Bmp3 signaling in OMSC osteogenic differentiation, we investigated whether Bmp3 knockdown rescued the perturbed osteogenic differentiation induced by miR-206-3p inhibitor. The results showed that at both the mRNA and protein levels, the altered expressions of Bmp3, Opn, Runx2, and Osx after miR-206-3p inhibition could be partly reversed by *Bmp3* knockdown **(Figure 4G, S2J)**. Furthermore, ALP and ARS assays showed that the decrease in mineralization caused by miR-206-3p inhibitor was rescued by Bmp3 knockdown **(Figure 4H)**.

In general, these results suggest that miR-206-3p is an important downstream factor of GATA4 that controls Bmp3 signaling, ultimately leading to OMSC osteogenic differentiation.

### Exosomal miR-206-3p from OMSCs regulates osteoclast activity by targeting NFATc1

**Figure 5:**
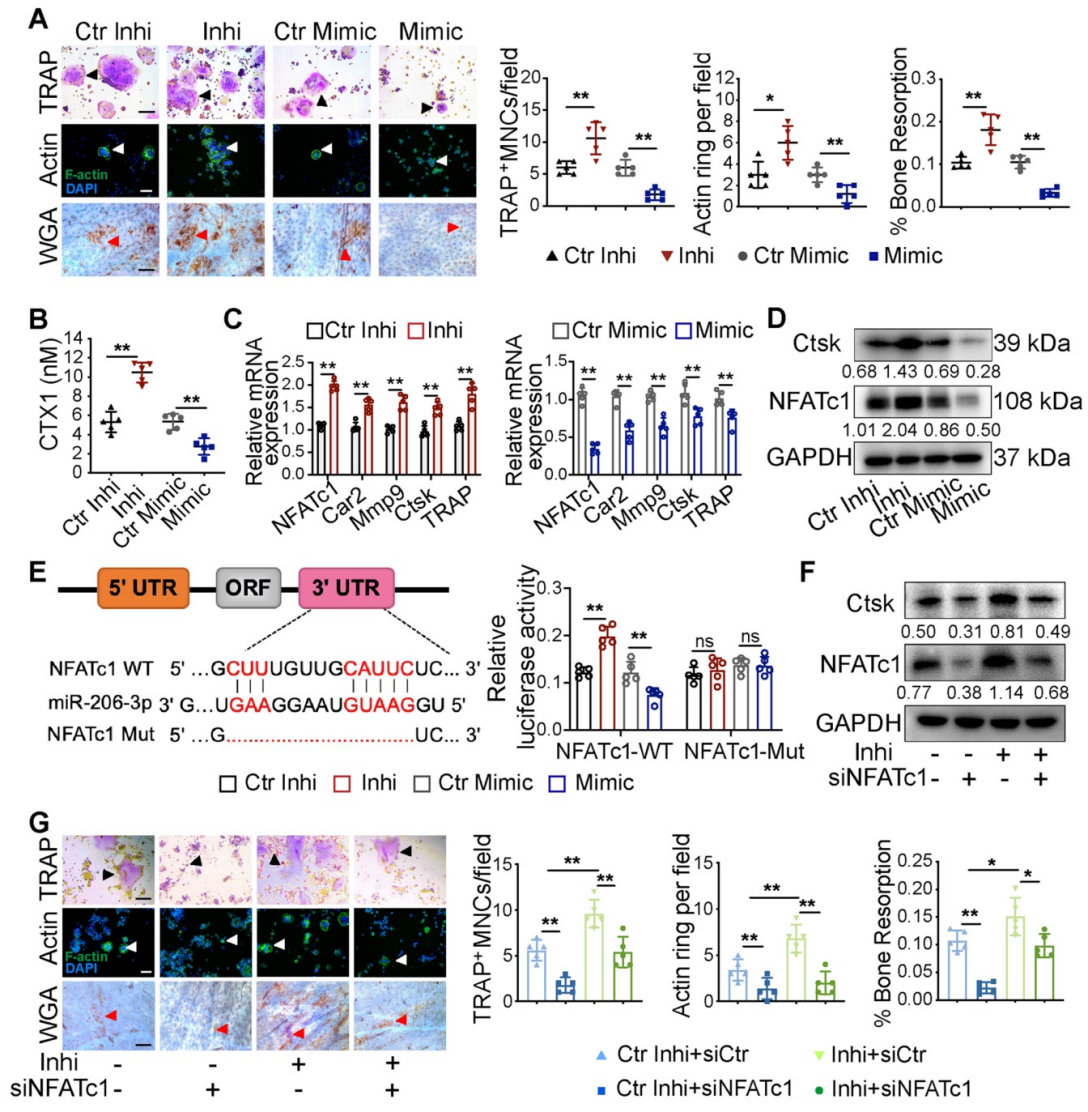
** Exosomal miR-206-3p from OMSCs regulates osteoclast activity by targeting NFATc1.** (A) The exosomes from OMSCs were treated with miR-206-3p inhibitor or mimic, respectively, and then co-cultured with RANKL-treated RAW264.7 cells. TRAP staining (black arrows), F-actin ring number per field (white arrows), and bone resorption pits (red arrows) using WGA (wheat germ agglutinin) staining were used to detect osteoclast formation and bone resorption (n = 5). Scale bar, 200 μm. (B) The protein level of carboxy-terminal collagen cross-links (CTX1) from the culture medium in (A) were assessed by ELISA (n = 5). (C) qRT-PCR was carried out to measure the mRNA levels of NFATc1, Car2, Mmp9, Ctsk and TRAP in osteoclasts in presence of OMExos transfected with miR-206-3p inhibitor or mimic (n = 5). (D) The protein levels of NFATc1 and Ctsk were detected by western blotting in osteoclasts treated with exosomes of miR-206-3p inhibitor or mimic (n = 5). (E) Identification of miR-206-3p binding sites on the 3' UTR of NFATc1. Dual-luciferase reporter assays were carried out to verify that miR-206-3p directly targeted the 3'UTR of NFATc1 (n = 5). (F) RANKL treated RAW264.7 cells were co-transfected with miR-206-3p inhibitor and siNFATc1, the protein levels of NFATc1 and Ctsk were examined by western blotting (n = 5). (G) RANKL treated RAW264.7 cells were co-transfected with miR-206-3p inhibitor and siNFATc1. TRAP staining (black arrows), F-actin ring number per field (white arrows), and bone resorption pits (red arrows) using WGA staining were used to detect osteoclast formation and bone resorption (n = 5). Scale bar, 200 μm. control inhibitor, Ctr-inhi; inhibitor, inhi; control mimic, Ctr-mimic. Ordinary one-way ANOVA. Each experiment was repeated at least three times with the same conditions. Data are shown as mean ± SD. ^*^P < 0.05, ^**^P < 0.01; ns, not significant.

To further verify the effect of miR-206-3p on exosome function in osteoclasts, exosomes from OMSCs were treated with miR-206-3p mimic or miR-206-3p inhibitor, and then co-cultured with RANKL-treated RAW264.7 cells. The knockdown and overexpression efficiencies of miR-206-3p in osteoclasts were examined by qRT-PCR **(Figure S3A)**. Formation of TRAP^+^MNCs, F-actin rings, and resorption pit area on bone slices were increased in the coculture after treatment with miR-206-3p inhibitor relative to control inhibitor **(Figure 5A, S3B)**. In comparison, the number of multinucleated osteoclasts and resorption pit area were decreased in the miR-206-3p mimic group **(Figure 5A)**. These results were corroborated by the increased release of CTX1 in the miR-206-3p inhibitor group and decreased release of CTX1 in the miR-206-3p mimic group **(Figure 5B)**. Consistent with the above results, qRT-PCR and western blotting analyses showed that the expressions of osteoclast-related genes were significantly increased in the coculture environment after miR-206-3p inhibitor treatment but repressed by treatment with the miR-206-3p mimic **(Figure 5C, D)**.

According to bioinformatics analysis using TargetScan, NFATc1, was predicted as the putative target gene of miR-206-3p. A dual-luciferase reporter assay further showed that cellular luciferase activity was markedly increased with miR-206-3p inhibition but decreased with miR-206-3p overexpression **(Figure 5E)**.

To investigate the expression of NFATc1 in osteoclast progenitors, we cultured bone marrow cells from WT and cKO mice in the presence of macrophage colony-stimulating factor (M-CSF) (20 ng/mL) for 3 d. The NFATc1 mRNA and protein levels were significantly higher in osteoclast progenitors from cKO mice compared with WT mice (**Figure S3C**). To further demonstrate the role of NFATc1 in the miR-206-3p-mediated repression of osteoclastogenesis, we investigated whether NFATc1 knockdown reverse the hyperactivity of osteoclastogenesis induced by miR-206-3p inhibitor. qRT-PCR analysis suggested that miR-206-3p inhibitor dramatically increased the mRNA levels of osteoclast-related genes **(Figure S3D)**. The protein levels of NFATc1 and Ctsk were also increased by miR-206-3p inhibitor, as shown by western blotting analysis. These effects of miR-206-3p inhibitor were partially reversed by NFATc1 knockdown **(Figure 5F)**. TRAP and F-actin staining indicated that the increased differentiation after miR-206-3p inhibition were partially reversed by NFATc1 deficiency **(Figure 5G)**. Consistent with the above results, the enhanced bone resorption caused by miR-206-3p inhibition was partly reversed by NFATc1 deficiency.

Therefore, these results indicate that miR-206-3p can regulate the differentiation and function of osteoclasts by targeting NFATc1.

### The role of GATA4-driven miR-206-3p in bone formation and bone resorption *in vivo*

**Figure 6:**
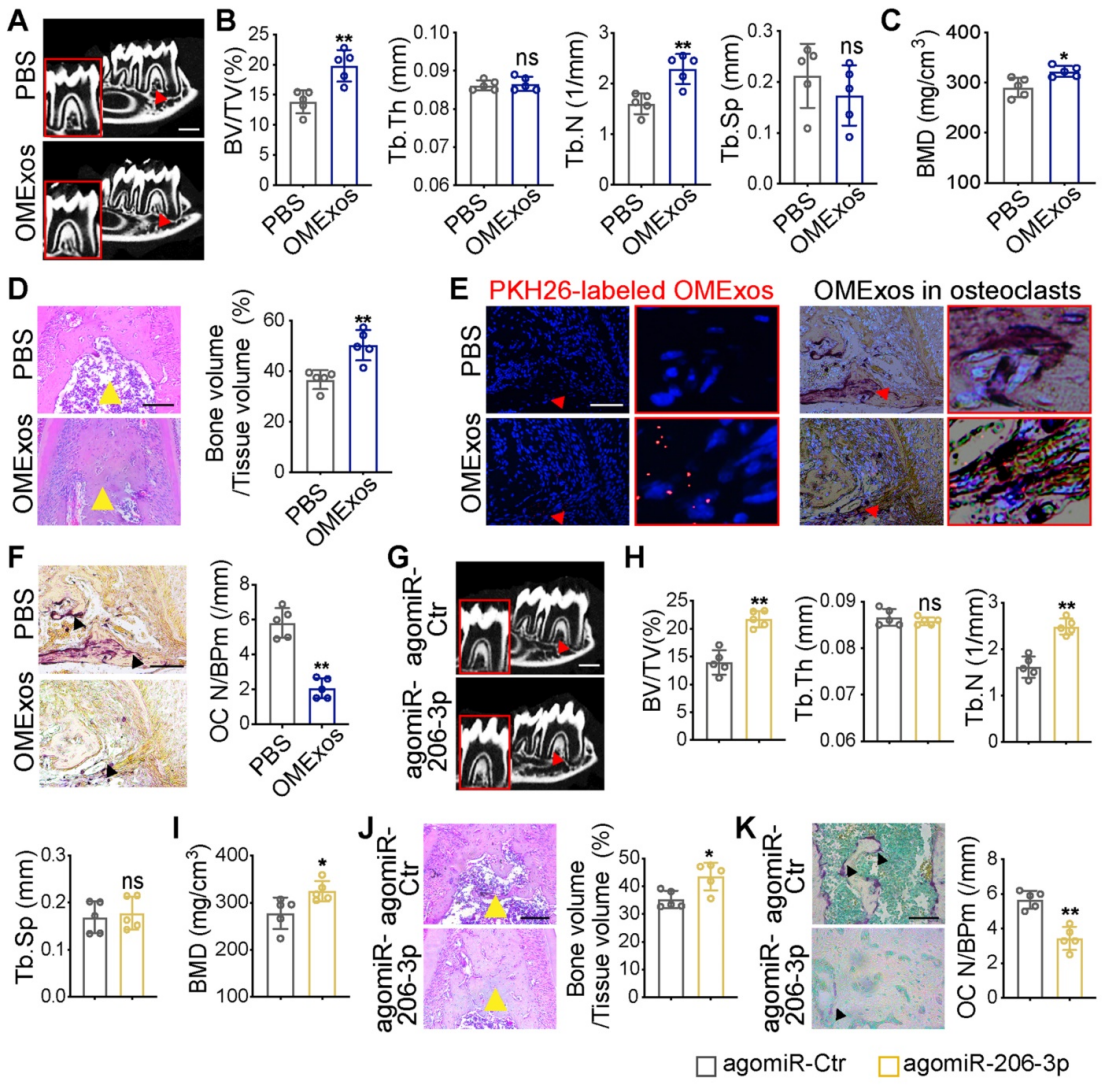
** Therapeutic effects of OMSCs-derived exosomes and agomiR-206-3p on bone metabolism in *Wnt1-cre;Gata4^fl/fl^* mice.** (A) *Wnt1-cre;Gata4^fl/fl^* (cKO) mice were locally injected into the buccal periosteum of the right mandibular first molar with vehicle (phosphate-buffered saline, PBS) or OMSCs-derived exosomes (OMExos) purified from *Gata4^fl/fl^* (WT) mice. Reconstructed 3D micro-CT images of the mandibles in different groups. Decreased mineralization area was denoted by red arrows (n = 5). Scale bars: 500 µm. (B) Micro-CT analysis of mandibles from the PBS and OMExos (n = 5). (C) Bone mineral density (BMD) of the alveolar bones in PBS or OMExos treated cKO mice (n = 5). (D) H&E staining identified bone mass (yellow arrows) at the bifurcation of the mandibular first molar root in PBS or OMExos treated cKO mice (n = 5). Scale bar: 200 µm. (E) Fluorescent microscopy and TRAP staining analysis revealing PKH26-labeled OMExos injected into bone marrow. Red arrow head indicated PKH26-labeled OMExos. Scale bars: 100 μm. (F) TRAP staining of osteoclasts in alveolar bone of mice treated with PBS or OMExos. Osteoclasts were denoted by black arrows. OC N/B.Pm (/mm), osteoclast number per bone perimeter. Scale bars: 100 μm. (G) cKO mice were locally injected into the buccal periosteum of the right mandibular first molar with agomiR-Ctr or agomiR-206-3p. Reconstructed 3D micro-CT images of the mandibles in different groups. Decreased mineralization area was denoted by red arrows (n = 5). Scale bars: 500 µm. (H) Quantification from G. BV/TV, bone volume/tissue volume; Tb.Th, trabecular thickness; Tb.N, trabecular number; Tb.Sp, trabecular separation (n = 5). (I) Bone mineral density (BMD) of the alveolar bones in agomiR-Ctr and agomiR-206-3p treated cKO mice (n = 5). (J) H&E staining identified bone mass (yellow arrows) at the bifurcation of the mandibular first molar root in agomiR-Ctr or agomiR-206-3p treated cKO mice (n = 5). Scale bar: 200 µm. (K) TRAP staining of osteoclasts in alveolar bone of mice treated with agomiR-Ctr or agomiR-206-3p. Osteoclasts were denoted by black arrows. Scale bars: 100 μm. Two-tailed Student's t test. Each experiment was repeated at least three times with the same conditions. Data are shown as mean ± SD. ^*^P < 0.05, ^**^P < 0.01; ns, not significant.

To investigate whether GATA4-driven miR-206-3p is a regulator of osteoblast and osteoclast activity, the *in vivo* therapeutic effects of OMExos or agomiR-206-3p on bone metabolism were evaluated in the *Wnt1-cre;Gata4^fl/fl^* mouse model. cKO mice were locally injected into the buccal periosteum of the right mandibular first molar with vehicle (phosphate-buffered saline, PBS), OMExos purified from WT mice, agomiR-Ctr, or agomiR-206-3p. Micro-CT analysis suggested that injection of OMExos from WT mice caused a remarkable increase in bone mass at the bifurcation of the mandibular first molar root **(Figure 6A-C)**. These results were consistent with H&E staining results **(Figure 6D)**. Incorporation of exosomes into cKO osteoclasts was confirmed using PKH26-labeling **(Figure 6E)**. Mice injected with OMExos from WT mice showed significantly lower osteoclast numbers than those in the control group **(Figure 6F)**.

To explore therapeutic value of miR-206-3p, we also constructed and delivered agomiR-206-3p into the buccal periosteum of the right mandibular first molar of cKO mice. Micro-CT analysis revealed greater trabecular bone mass in agomiR-206-3p treated cKO mice compared with agomiR-Ctr counterparts **(Figure 6G-I)**. Additionally, H&E staining revealed that a considerable amount of trabecular bone was retained in agomiR-206-3p treated cKO mice **(Figure 6J)**. Consistently, injection of agomiR-206-3p contributed to a greater decrease in osteoclast numbers in cKO mice **(Figure 6K)**. These results indicate that miR-206-3p had a vital role in the suppression of bone degeneration.

### Local knockdown of miR-206-3p inhibited bone formation and promoted bone resorption

**Figure 7:**
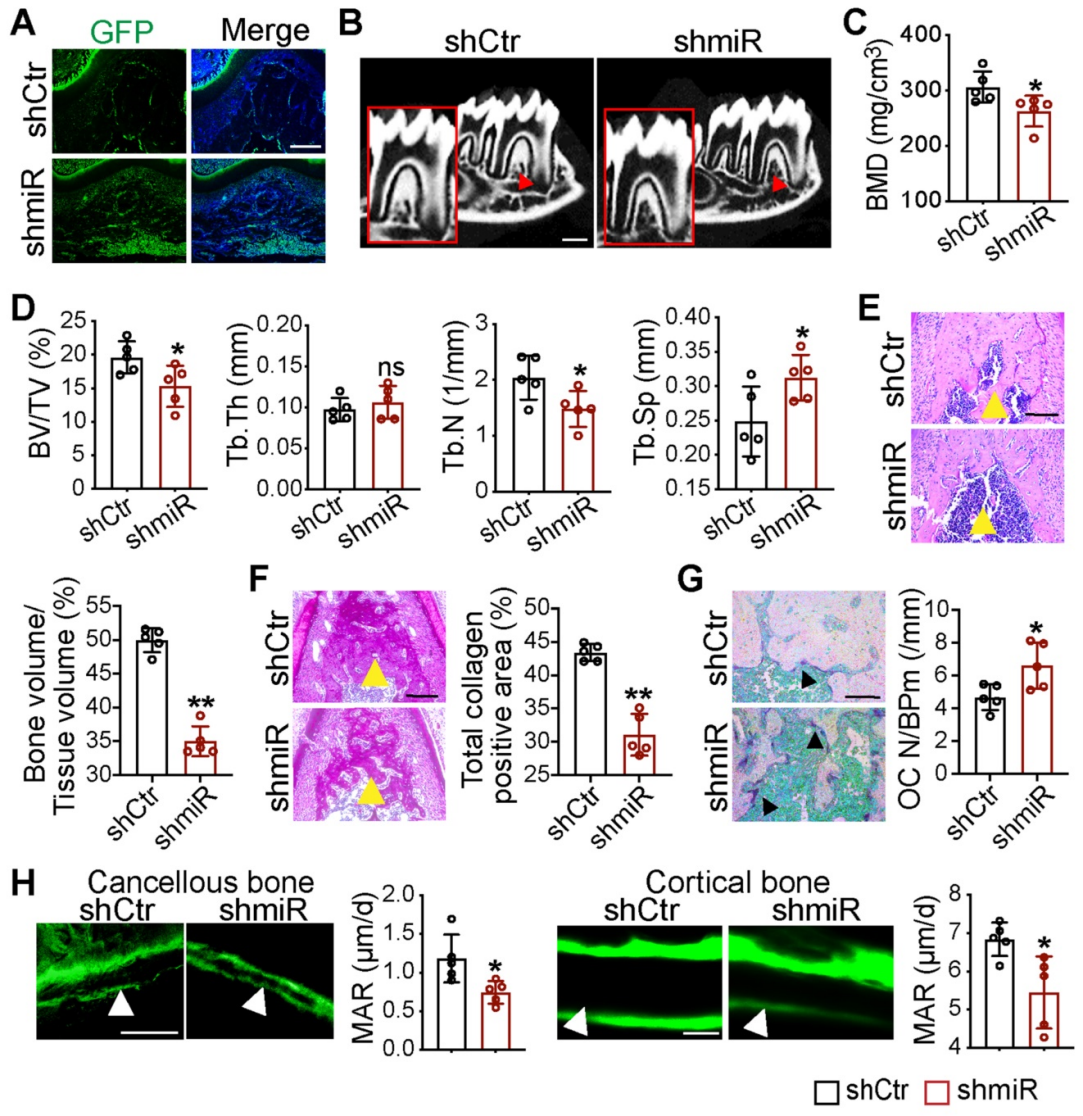
** Local knockdown of miR-206-3p inhibited bone formation and promoted bone resorption *in vivo*.** (A) Double-labeled fluorescent immunostaining of GFP (green) and DAPI (blue) stained the mandibles at 21 d. Scale bar: 200 µm. (B) Micro-CT reconstruction of mandibles from mice injected in GFP or miR-206-3p inhibitor lentivirus (shCtr or shmiR) (n = 5). Decreased mineralization area was denoted by red arrows. Scale bars: 500 µm. (C) Bone mineral density (BMD) of the alveolar bones in shCtr and shmiR mice (n = 5). (D) Quantification from B. BV/TV, bone volume/tissue volume; Tb.Th, trabecular thickness; Tb.N, trabecular number; Tb.Sp, trabecular separation (n = 5). (E) H&E staining identified bone mass (yellow arrows) at the bifurcation of the mandibular first molar root (n = 5). Scale bar: 200 µm. (F) Total collagen staining showed the bone mass (yellow arrows) of mandibles (n = 5). Scale bar: 200 µm. (G) Representative TRAP staining of the mandibles. Osteoclasts were denoted by black arrows (n = 5). OC N/B.Pm (/mm), osteoclast number per bone perimeter. Scale bar: 100 µm. (H) Calcein-double labelling assay showed new bone formation (white arrows) at mandibles (n = 5). Scale bar: 50 µm. Two-tailed Student's t test. Each experiment was repeated at least three times with the same conditions. Data are shown as mean ± SD. n = 5. ^*^P < 0.05, ^**^P < 0.01; ns, not significant.

To further examine the function of miR-206-3p in the development of maxillofacial bone, we created an animal model with miR-206-3p inhibited in the maxillofacial bone microenvironment. Lentiviruses that knocked down miR-206-3p (shmiR) or the control (shCtr) were injected into the buccal periosteum of the right mandibular first molar using a microinjector. The selected shCtr and shmiR vectors contained the GFP tag, and cells infected with the lentivirus expressed GFP. Indeed, strong GFP expression was observed in the shCtr and shmiR groups **(Figure 7A)**. Micro-CT analysis suggested that mice in the shmiR group showed a remarkable decrease in bone mass at the bifurcation of the mandibular first molar root **(Figure 7B-D)**. Notably, H&E and total collagen staining showed that the miR-206-3p inhibitor decreased trabecular bone structure **(Figure 7E, F)**. In addition, mice injected with lentivirus containing the miR-206-3p inhibitor showed significantly higher osteoclast numbers than those in the control group **(Figure 7G)**. To further confirm the decreased osteoblast activity in the shmiR group, calcein double labeling was carried out to measure the mineral apposition rate (MAR). The results indicated that MAR was reduced in mice in the shmiR group** (Figure 7H)**. The above data indicate that lentivirus containing a miR-206-3p inhibitor prevents bone formation and promotes bone resorption in mandible tissues.

## Discussion

The clinical pathology and pathogenesis of craniofacial disorders are related to orofacial hypoplasia. In the process of orofacial bone development, the coordination of OMSC-derived osteoblasts and osteoclasts can maintain the dynamic balance of bone remodeling. A deeper understanding of the interaction between NCC-derived OMSCs and cells from other tissues may help to identify potential therapeutic targets for orofacial dysplasia. In the present study, we defined a novel signaling axis, GATA4-miR-206-3p-Bmp3, in OMSC-mediated osteogenic differentiation. We also suggest that GATA4 functions as an orofacial bone development promotor and affects osteoclastogenesis via OMSC-derived miR-206-3p-containing exosomes by regulating NFATc1. In summary, this study explains the mechanism of orofacial bone development from the perspective of OMSC-induced bone formation and the crosstalk between OMSCs and osteoclasts through exosome-mediated miRNA transfer.

In this study, we found that the number of osteoclasts that originated from the mesoderm in the orofacial bone of *Wnt1-cre;Gata4^fl/fl^* mice was increased compared to that of WT mice. Moreover, CTX1 was significantly increased and the P1NP remained unaltered in the serum of cKO mice, which indicated the stimulation of bone resorption after GATA4 deletion. These results suggest that the low bone mass in *Wnt1-cre;Gata4^fl/fl^* mice is due to increased bone turnover. We propose the presence of crosstalk between OMSCs and osteoclasts in the orofacial bone microenvironment. However, osteoblast differentiation from *Wnt1-cre;Gata4^fl/fl^*precursors was decreased *in vitro*, which is contradictory to the *in viv*o P1NP ELISA assay. The functions of osteoblasts are determined by various factors within the complex bone microenvironment, which are much different from the *in vitro* assay. It is impossible to rebuild the intricate bone microenvironment in osteogenic cultures. This may explain the discrepancy between the P1NP ELISA results of *in vivo* and the osteoblast differentiation *in vitro* assays.

In this study, we demonstrated that miR-206-3p was significantly downregulated in shGATA4 OMSCs and their supernatant-derived exosomes. miR-206-3p is widely acknowledged as a characteristic, positive regulator of muscle differentiation 27 and is also expressed at remarkable levels in brown adipocytes 28. Studies also indicated the potential role of miR-206-3p in the pathogenesis and pathology of osteoporosis 21, 22. However, the specific mechanism of miR-206-3p in bone remodeling was not evaluated. In our study, we found that miR-206-3p is an important downstream factor of GATA4, and GATA4-miR-206-3p-Bmp3 signaling plays a positive role in osteogenic differentiation. Previous studies have proven that Bmp3 is the most abundant Bmp in bone. *Bmp3^-/-^* mice were shown to have twice as much trabecular bone as WT mice, while mice overexpressing Bmp3 generated spontaneous rib fractures 26, 29, 30. *In vitro*, overexpression of Bmp3 inhibited osteoblast differentiation, while loss of Bmp3 promoted osteoblast and osteocyte colony formation 25. In line with these results, our results from both inhibition and gain-of-function approaches suggest that miR-206-3p promotes osteogenic differentiation by negatively regulating Bmp3 expression.

Previous reports have elucidated the direct or indirect role of NFATc1 in miRNAs-regulated osteoclastogenesis. Gain-of-function of miR-30a or miR-7b was shown to represses RANKL-induced osteoclast differentiation by targeting the NFATc1 pathway 31, 32. By posttranscriptional gene silencing of NFATc1, miR-124 was reported to diminish osteoclast differentiation 33, 34. In the present research, exosomal miR-206-3p derived from OMSCs inhibited osteoclast differentiation by targeting the 3'UTR of NFATc1. Notably, inhibition of NFATc1 averted the stimulative effect of miR-206-3p inhibitor on osteoclastogenesis, suggesting that miR-206-3p inhibited osteoclast differentiation in NFATc1-dependent manner.

Bone resorption also is locally regulated by the balance between RANKL and OPG 35. RANKL is crucial to induce osteoclast differentiation. OPG is a decoy receptor for RANKL and negatively regulates RANKL binding to RANK. We found that miR-206-3p knockdown did not affect OPG but enhanced the ratio of RANKL to OPG during the differentiation of OMSCs. These results suggest that GATA4-miR-206-3p axis in OMSCs could inhibit RANKL expression and may be also involved in osteoclast differentiation. Thus, GATA4-miR-206-3p axis in OMSCs may bidirectionally regulate osteoclast differentiation by both OMExos and RANKL/OPG signaling. How the RANKL/OPG signaling in OMSCs impacts the function of osteoclasts thus remains to be further determined.

Considering the inhibitory role of exosomal miR-206-3p derived from OMSCs on osteoclastogenesis, we further evaluated the *in vivo* role of GATA4-driven miR-206-3p signatures in bone remodeling. We assessed the therapeutic effect of exosomal miR-206-3p by locally administering OMExos or agomiR-206-3p to cKO mice. We revealed that OMSCs-derived exosomes from WT mice transferred into osteoclasts and significantly decreased osteoclastogenesis which was attributed to the high level of miR-206-3p. Further, we explored the function of chemically modified small RNAs of agomiR-206-3p in bone marrow. These RNAs decreased bone resorption and increased bone formation in cKO mice, while similar experiments in WT mice with shmiR-206-3p produced opposite effects. All these results suggest the positive role of GATA4-miR-206-3p signaling in bone formation and that exosomal miR-206-3p could serve as an intercellular messenger to mediate OMSC-to-osteoclast communication for inhibiting osteoclastic bone resorption.

Similar to our *in vivo* results, previous studies also showed that bone metabolism can be regulated by exosomal-containing miRNAs extracted from MSCs 14, 36. Growing evidence suggests the therapeutic benefits of MSC-derived exosomes for diseases such as osteoarthritis and rheumatoid arthritis 37. Compared to MSCs in transplantation therapies, exosomes are smaller, so they can circulate more easily, and they are much easier to obtain 38. Their usage avoids the transfer of major histocompatibility complex (MHC) class I proteins or potential mutated DNA from cells 38. However, there are still some challenges to pharmaceutically characterizing exosomes because of their complex structure. Therefore, although exosomes might represent a novel, promising therapeutic treatment for the future, we still need further investigation for their applications in bone diseases.

Taken together, these findings support the view that OMSCs not only directly participate in orofacial bone development by differentiating into osteoblasts but also indirectly participate in orofacial bone development by regulating the fate of osteoclasts. Our research reveals the mechanism of orofacial bone development more accurately, comprehensively and systematically than previous studies, providing a new direction for further exploration in this field.

## Materials and Methods

### Mice

*Wnt1-Cre* and *Gata4^fl/fl^* mice have been described 24. To obtain mice with GATA4-deficient NCCs, we crossed *Wnt1-Cre;Gata4^fl/+^* males with *Gata4^fl/fl^* females (C57/BL6). *Gata4^fl/fl^* male mice and *Wnt1-Cre* male mice were used as WT mice and *Wnt1-Cre;Gata4^fl/fl^*male mice were used as cKO mice. The mice were obtained from the Model Animal Research Center of Nanjing University (MARC). All experiments were carried out with the approval of the Ethics Committee of the Stomatological School of Nanjing Medical University (IACUC-2012036).

### Micro-computed tomography (micro-CT) analysis

WT and cKO mice were collected at the P21 and P56, and heads were fixed in 4% paraformaldehyde (PFA) overnight. Scanning was performed using a micro-CT scanner (Skyscan, Belgium) 39. Finally, 3D micro-CT reconstruction of the skull was acquired and analyzed using NRecon v1.6 and CTAn v1.13.8.1 software. Bone structures were analyzed by the following parameters: bone volume/tissue volume (BV/TV), trabecular thickness (Tb.Th), trabecular number (Tb.N), and trabecular separation (Tb.Sp).

### Tissue Preparation and Histology Stains

Heads of mice were harvested and fixed in 4% PFA overnight. Samples were decalcified in 10% EDTA for 2-4 weeks and embedded in paraffin. Then samples were sectioned at 5 µm and mounted on glass slides 40. Histological analysis was performed including staining with hematoxylin/eosin (H&E) and total collagen (1% Sirius Red), using paraffin sections. The protocol was performed as described previously 16, 41.

For tartrate-resistant acid phosphatase (TRAP) staining, sections (5 µm) were stained using the TRAP Kit (Sigma, 387A-1KT) following the manufacturer's instructions. Data are included as graphs of osteoclast number per bone perimeter (OC N/B.Pm).

### Culture of OMSCs and osteoclasts

The culture of orofacial mesenchymal stem cells (OMSCs) was performed following the established protocol 1, 3. Mandibles were dissected from 8-week-old male mice, chopped into pieces, and digested with 2 mg/mL collagenase type 1 (Sigma, USA) and 4 mg/mL dispase II (Sigma, USA) at 37 ℃ for 60 min. After counting viable cells, 1.5 × 10^7^ cells were plated into a 100 mm dish. The primary cells from mandibles were applied for analysis after 3 - 4 passages and cultured in DMEM supplemented with 10% fetal bovine serum (FBS) and 1% penicillin/streptomycin. To characterize the OMSC markers, Sca-1 (stem cell antigen-1; Abcam, ab51317), CD73 (proteintech, 12231-1-AP) were incubated by immunocytochemistry.

Primary bone marrow-derived macrophages (BMMs) were extracted from tibias and femurs of WT and cKO male mice (4 weeks). The flushed cells were cultured in α-minimum essential medium (α-MEM) for 3 h to remove adherent cells, and non-adherent cells were seeded into new plates and cultured in α-MEM with M-CSF (20 ng/mL, Sigma) for 3 d, resulting in osteoclast progenitors.

Mouse mononuclear macrophages cell line, RAW264.7 cells, were purchased from the Wuhan Procell Biological Technology Co. (Wuhan, China). To generate osteoclasts, RAW264.7 cells (5 × 10^4^ cells/well) were cultured in 10% DMEM containing 20 ng/mL RANKL (Sigma, USA). The complete medium was changed every 2 d.

### TRAP staining, F-actin staining, and bone resorption pit assay

RAW264.7 cells were seeded into 24-well plates (5 × 10^4^ cells/well) and treated with 20 ng/mL RANKL. After 4 d, cells were fixed in 4% PFA for 10 min and incubated with TRAP staining solution according to the manufacturer's instructions (Sigma, 387A-1KT). Three or more than three TRAP-positive multinucleated cells (TRAP+ MNCs) were considered as osteoclasts.

For F-actin ring staining, RAW264.7 cells were seeded into 24-well plates (5 × 10^4^ cells/well) and treated with 20 ng/ml RANKL. After 4 d, cells fixed with 4% PFA for 10 min and stained with 7 uL phalloidin for 30 min (AmyJet, AMJ-KT0003). DAPI at 1:1000 was used to stain Nuclei. Fluorescence images were observed under fluorescence microscope (Leica Microsystems, Germany).

For the bone resorption pit assay, RAW264.7 cells were seeded on bovine bone slides to differentiate into osteoclasts. After 6 d, bone slices were washed with PBS and then incubated with 20 μg/mL wheat germ agglutinin (Sigma, USA) for 40 min. Afterward, the bone slices were incubated in diaminobenzidine (DAB) and visualized with the Leica Application Suite (Leica, Germany). The results were presented as a percentage of the total plate area.

### ELISA assay

For the serum P1NP and CTX1 assay, the blood of 8-week-old male mice was collected under anesthesia. For the medium ELISA assay, osteogenic culture supernatants were collected, centrifuged at 4000 g at 4 °C, and frozen at -80 °C until used. Serum and supernatants CTX1 were determined using RatLAPS EIA kit (Immunodiagnostics Systems, UK). P1NP concentration was determined with the mouse P1NP ELISA Kit (Immunoway, #KE1744). RANKL and OPG were detected using R&D Systems ELISA kits (Minneapolis, USA). The protocol was performed according to the manufacturer's instructions.

### Isolation and identification of exosomes

Exosomes were collected according to the established protocol 42. OMSCs were cultured in exosome-depleted FBS (System Biosciences, USA) 43. After 48 h, the supernatant of OMSCs was collected. To isolate the exosomes of OMSCs, the supernatant was centrifuged at 300 g for 10 min, 2000 g for 10 min, and 10000 g for 30 min to remove cell debris. Subsequently, the reminding supernatant was centrifuged 100000 g for 70 min twice to remove the contaminating protein. The final pellets were resuspended in 100 µL PBS and stored at -80 ℃.

The nanoparticle tracking analysis (NTA) was performed to determine the size distribution of OMSCs-derived exosomes (OMExos) by ZetaView Nanoparticle Tracking Analyzer (PMX, Germany). Exosome morphologies were imaged under a transmission electron microscope (Hitachi, Japan). Antibodies against HSP70 (Cell Signaling, #4872, 1:1000) and CD63 (Abcam, ab217345), Calnexin (proteintech, #10427-2-AP, 1:1000) proteins were used to analyze the protein into exosomes by western blotting analysis.

### Uptake of PKH26-labeled OMExos

Exosomes derived from OMSCs were labeled with PKH26 (Sigma, USA) according to the manufacturer's instructions. Briefly, PKH26 (2 μL) was mixed with Diluent C (500 μL). Then, 500 μL Diluent C including OMExos were added into the mixture and incubated at room temperature (RT) for 3 min. Subsequently, 0.5% exosome-depleted FBS (1 mL) (System Biosciences [SBI], USA) was incubated at room temperature for 5 min to stop the reaction. Then, supernatant containing OMExos was harvested and incubated with RAW264.7 cells in presence with 20 ng/mL RANKL at 37 ℃ for 0, 6, 12, 24 h respectively. Finally, cells were stained with DAPI for 1 min after being fixed with 4% PFA for 10 min and visualized under a fluorescence microscope (Leica Microsystems, Germany).

### Osteoclast viability and mobility assays

To evaluate the viability of RAW264.7 cells, cell counting kit-8 (CCK-8) assays were performed according to the manufacturer's instructions. Briefly, the cells were seeded in 96-well plates in the presence of OMExo-G4 or OMExos-Ctr for up to 72 h. After incubating the cells with CCK-8 solution for 3 h, the 96-well plates were placed in a microplate reader and were measured at 450 nm.

To evaluate the mobility of RAW264.7 cells, cells were cultured in an osteoclastogenic medium in the presence of OMExo-G4 or OMExos-Ctr for 24 h. Wounds were inflicted using a pipette tip when cells grew to 95-100%. Then, plates were washed with a growth medium to remove the floating cells. Wound healing was cultured for 0, 12, and 24h, and then photographed.

### Transfection of GATA4 lentivirus, miR-206-3p inhibitor and mimic

Lentivirus of shRNA to knockdown GATA4 expression (shGATA4; 5'-CCAAGCAGGACTCTTGGAA-3') and negative control (shCtr; 5'-TTCTCCGAACGTGTCACGT-3') were synthesized by GenePharma (GenePharma, China). OMSCs were seed in a 100 mm dish at a density of 7 × 10^6^. When the cells reached 60% - 70% confluence and then infected with the lentivirus (multiplicity of infection = 50) in the presence of 10 µg/mL Polybrene. The medium was changed after 24 h.

Control inhibitor, miR-206-3p inhibitor, control mimic, or miR-206-3p mimic (50 nM, final concentration) were transfected into cells by using Lipofectamine 2000 (Invitrogen, USA). Cells were harvested for analyses at least 48 h after transfection.

### Co-culture of OMSCs and exosomes with osteoclasts

The OMSCs from WT or cKO mice were plated in the basolateral chamber of the 24-well transwell system (5 × 10^4^ cells/well) while the M-CSF and RANKL-treated BMMs were placed in the apical chamber. The BMMs in the apical chamber were stained using TRAP staining solution to detect osteoclastogenesis.

The exosomes from OMSCs were treated with miR-206-3p mimic and miR-206-3p inhibitor, respectively (50 nM, final concentration), and then co-cultured with RANKL-treated RAW264.7 cells in 24-well plates for 48 h. The miR-206-3p expression was determined using qRT-PCR.

### RNA extraction and miRNA microarray analysis

Total exosome RNA was extracted with the Total Exosome RNA & Protein Isolation Kit (Life Technologies) according to the manufacturer's instructions. Microarray analysis was done by using the Multispecies miRNA 4.0 Array (Affymetrix GeneChip, USA), and each group had three biological replicates. Labeling and hybridization were conducted according to the manufacturer's instructions. GeneChips were washed and stained in the Affymetrix Fluidics Station 450. GeneChips were scanned on a GeneChip Scanner 3000 7G instrument (Affymetrix). The raw data were preprocessed with Robust Multichip Analysis (RMA) algorithm to perform the background correction. Threshold for differentially expressed miRNAs between two groups was set as a fold change >1.5; p<0.05.

### Differentiation of OMSCs and ARS and ALP activity assay

To induce osteogenic differentiation, OMSCs were cultured in an osteogenic medium supplemented with 50 µM ascorbic acid, 10^-7^ M dexamethasone and 10 mM β-glycerophosphate. Osteogenic differentiation medium was changed every 3 d.

OMSCs were cultured in osteogenic medium for 14 d, and the calcified nodule was measured by Alizarin Red (Beyotime, China) for 30 min at room temperature. Calcified nodules were eluted by 10% cetylpyridinium chloride (CPC) and calcium mineral content was analyzed at 562 nm. Cells were cultured in the mineralization-inducing medium for 5 d and stained with a BCIP/NBT alkaline phosphatase color development kit (Beyotime Institute of Biotechnology, China).

### Quantitative Real-time PCR analysis (qRT-PCR)

Total RNA was extracted from cells by Trizol reagent according to the manufacturer's instructions. The mRNA was employed to generate cDNA through HiScript^®^ II Q RT SuperMix for qPCR (Vazyme, China) according to the manufacturer's recommendations. Exosomal miR-206-3p was extracted by using miRNeasy Serum/Plasma kit (QIAGEN, USA). miR-206-3p expression was detected through the All-in-One miRNA qRT-PCR Detection Kit (GeneCopoeia, USA). Levels of each miRNA or mRNA were normalized to the U6 or GAPDH levels. The primers used are listed in Supplemental Table S1. To normalize miRNA expression, the miR-206-3p levels of exosomes were compared to spiked-in ce-miR-39, which was used as the reference by using the miRNeasy Serum/Plasma Spike-In Control kit (QIAGEN, USA).

Each experiment was performed three times. Data were quantified using the 2^-∆∆CT^ method.

### Western blotting

Proteins were collected by using a cell lysis reagent containing the protease inhibitor 44. Equal amount of protein was loaded and separated on a 10% SDS-PAGE gel and then transferred to 0.22-µm PVDF membranes. Membranes were blocked with 5% BSA for 2 h and incubated with primary antibodies at 4 °C overnight. After being incubated with the secondary antibodies for 1 h at room temperature, membranes were washed three times in Tris-buffered saline with Tween-20 and visualized by chemiluminescence. Semiquantitative measurements were carried out by ImageJ pro software.

The following primary antibodies were used for western blotting. HSP70 (Cell Signaling, #4872, 1:1000), CD63 (Abcam, #ab217345, 1:1000), Calnexin (proteintech, #10427-2-AP, 1:1000), NFATc1 (Santa Cruz, #sc7294, 1:1000), Ctsk (Santa Cruz, #sc-48358, 1:100), GAPDH (Bioworld, #AP0063, 1:5000), Opn (Abcam, #ab63856, 1:1000), Ocn (Abcam, #ab93876, 1:1000), Runx2 (Abcam, #ab76956, 1:1000), Osx (Abcam, #ab22552, 1:1000), Bmp3 (Immunoway, #YT0499, 1:2000).

### Dual-luciferase reporter assay

For one part, 293T cells were seeded in 24-well plates (2×10^5^ cells per well). When cells grew to 60% - 70%, they were infected with lentivirus of control inhibitor/mimic, miR-206-3p inhibitor/mimic, pGL3-basic reporter vector (Promega, USA) containing the 3' UTR fragment of NFATc1 or Bmp3, respectively.

For another part, cells were transfected with shCtr and pEZX-miR-206 or shGATA4 and pEZX-miR-206 promoter-luciferase by using Lipofectamine 2000 (Invitrogen, USA). After 48 h transfection, luciferase activities were measured by the Dual-Luciferase Reporter Assay Kit (Vazyme, China).

### Lentivirus injection and therapeutic administration in *Wnt1-cre;Gata4^fl/fl^* mice model

Male mice (C57/BL6) in the experimental group received 6 µL lentiviral supernatant (miR-206-3p inhibitor, shmiR-206-3p; 1 × 10^9^ TU/mL) injected in the buccal periosteum of the right mandibular first molar, while mice of the control group administered the same dose of control lentivirus (control inhibitor, shCtr).

Control *Wnt1-cre;Gata4^fl/fl^*male mice received a local injection of 10 µL PBS or agomiR-Ctr (1 μM), while experimental mice were instead injected equivalent volumes of OMExos (2.0 mg/mL) from WT mice or agomiR-206-3p (1 μM). Specially labeled and chemically modified miRNA agomiR is stable and can maintain activity in bone marrow environments for at least 2 weeks. Two injections were added every 3 d until mice at P21 45. At the end of the experiment, bone samples were analyzed as above.

### Calcein double labeling

Mice were given calcein (30 mg/kg with body weight) at 17 d and 3 d by intraperitoneal injections before euthanasia 46. Mouse heads were detached and fixed in 70% alcohol for 24 h. After gradient dehydration, undecalcified mouse heads were embedded in destabilized methyl methacrylate (MMA) according to standard protocols and the tissue blocks were cut at 10 µm thickness.

### Statistical analysis

All experiments in this study were carried out in triplicate to ensure reliability. Results were expressed as the means ± Standard Deviation (SD). Statistical significance of two comparisons comparison was performed for Student's t-test. Analysis across multiple groups was assessed using one-way ANOVA. P-value < 0.05 was considered statistically significant.

## Supplementary Material

Supplementary figures and tables.

## Abbreviations

OMSCs: orofacial mesenchymal stem cells
BMMs: bone marrow-derived macrophages
NCCs: neural crest cells
TEM: transmission electron microscopy
NTA: nanoparticle tracking analysis
OMExos: OMSCs-derived exosomes
TRAP: tartrate-resistant acid phosphatase
WGA: wheat germ agglutinin
ARS: Alizarin red staining
ALP: alkaline phosphatase
miRNA: microRNA
qRT-PCR: quantitative real-time PCR
Bmp3: bone morphogenetic protein-3
NFATc1: nuclear factor of activated T-cells cytoplasmic
Car2: carbonic anhydrase II
Mmp9: matrix metalloproteinase-9
Ctsk: Cathepsin K
Opn: osteogenic genes including osteopontin
Ocn: osteocalcin
Runx2: runt-related transcription factor 2
Osx: osterix
MAR: mineral apposition rate
