# Supplementary material for: GATA4-driven miR-206-3p signatures control orofacial bone development by regulating osteogenic and osteoclastic activity
Source: Theranostics. 2021 Jul 25;11(17):8379–95. doi: 10.7150/thno.58052 (PMC8344011; doi:10.7150/thno.58052)
Supplement: Supplementary file 1 — Supplementary figures and tables. [file thnov11p8379s1.pdf]

Supplementary Figures

GATA4-driven miR-206-3p signatures control orofacial bone development by regulating osteogenic and osteoclastic activity

Shuyu Guo[2, 3, 4*], Jiawen Gu[3, 4*], Junqing Ma[2, 3, 4*], Rongyao Xu[3, 4], Li Meng[3, 4], Haojie Liu[3, 4], Qingheng Wu[3, 4], Lu Li[1, 3, 4] & Yan Xu[1, 3, 4 ✉]

[1] Department of Periodontology, The Affiliated Stomatological Hospital of Nanjing Medical University, Nanjing, China.

[2] Department of Orthodontics, The Affiliated Stomatological Hospital of Nanjing Medical University, Nanjing, China.

[3] Jiangsu Key Laboratory of Oral Diseases, Nanjing Medical University, 140 Hanzhong Road, Nanjing 210029, China.

[4] Jiangsu Province Engineering Research Center of Stomatological Translational Medicine, Nanjing, China.

*These authors contributed equally to this work.

✉ Correspondence author: Yan Xu, Nanjing Medical University, 140 Hanzhong Road, Nanjing 210029, China. E-mail: yanxu@njmu.edu.cn, Fax: 0086-25-85031976

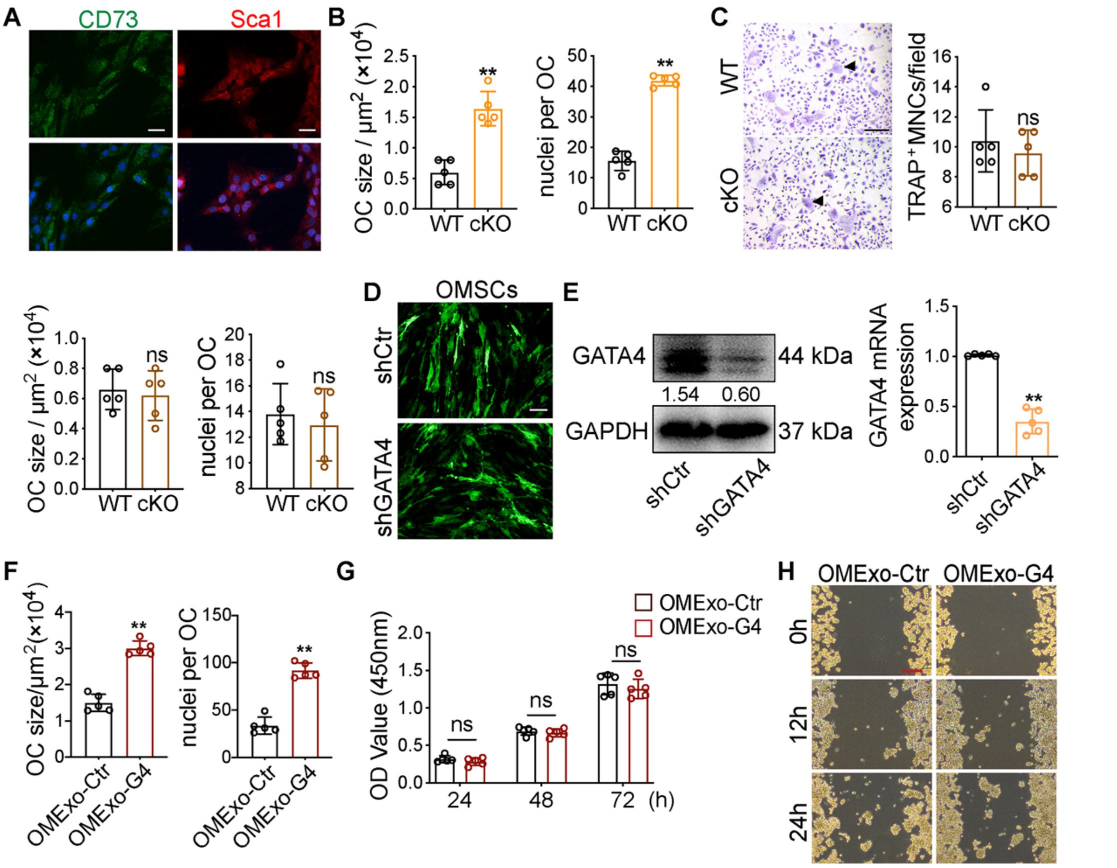

Figure S1. Exosomes derived from OMSCs promote osteoclastic differentiation and function.

(A) Images of OMSCs stained with DAPI to identify nuclei, and immunostained for the mesenchymal stem cell-associated markers (CD73 and SCA1). Scale bar: 200 μm. (B) The size and the nuclei numbers of TRAP-positive multinucleated cells ($TRAP^{+}$MNCs) in Figure 2A were measured (n = 5). (C) M-CSF and RANKL treated bone marrow-derived macrophages (BMMs) from $Gata4^{fl/fl}$ (WT) mice and *Wnt1-cre*;$Gata4^{fl/fl}$ (cKO) mice, and then the cells were stained for TRAP. The size and the nuclei numbers of $TRAP^{+}$MNCs were measured (n = 5). Scale bar: 200 μm. (D) OMSCs infected with lentivirus was assessed by fluorescence microscopy. Scale bar: 200 μm. (E) Efficiency of GATA4 knockdown in the protein and mRNA level after infection with lentivirus and quantitative analysis of western blotting and

qRT-PCR analysis (n = 5). (F) The size and the nuclei numbers of TRAP$^+$MNCs in Figure 2G were measured (n = 5). (G) RAW264.7 cells were cultured in a medium with exosomes isolated from shCtr or shGATA4 OMSCs (OMExo-Ctr, OMExo-G4). After 24 h, cell viability was measured by CCK-8 assay (n = 5). (H) RAW264.7 cells were cultured in osteoclastogenic medium in the presence of OMExo-Ctr or OMExo-G4. Cell mobility was measured by wound healing assay. Scale bar: 200μm. Two-tailed Student's t test. Each experiment was repeated at least three times with the same conditions. Data are shown as mean ± SD. $^{**}P < 0.01$; ns, not significant.

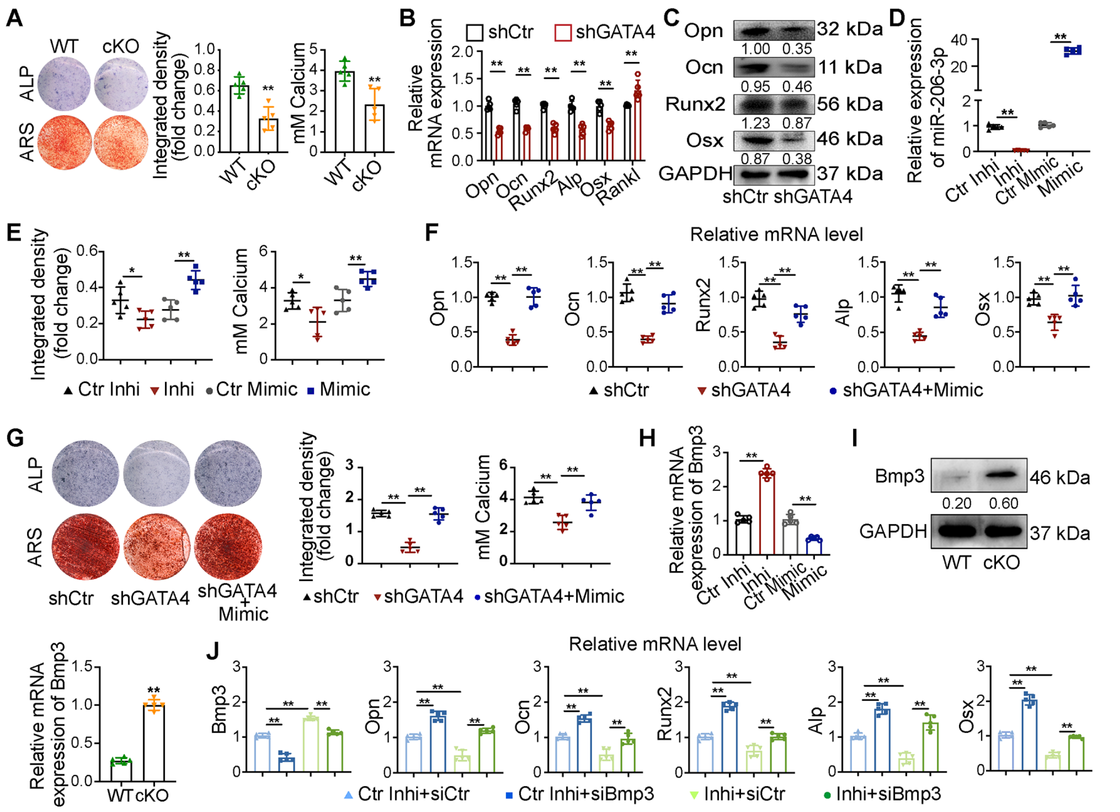

Figure S2. GATA4-miR-206-3p-Bmp3 signaling in regulating osteogenic differentiation of OMSCs.

(A) The ALP activity and the number of mineral nodules of OMSCs from *Gata4*$^{fl/fl}$ (WT) mice or *Wnt1-cre;Gata4*$^{fl/fl}$ (cKO) mice were accessed by ALP and ARS assay (n = 5). Two-tailed Student's t test. (B) Indicated osteogenic gene expression analysis of shGATA4-transfected OMSCs as compared with shCtr OMSCs after 5 days following mineralization induction (n = 5). Two-tailed Student's t test. (C) Protein expression levels of several osteogenic markers in shGATA4-transfected OMSCs were detected by western blotting as compared with shCtr OMSCs after 5 days following mineralization induction (n = 5). Two-tailed Student's t test. (D) qRT-PCR analysis showed the knockdown and over expression efficiency of miR-206-3p in OMSC-derived osteoblasts (n = 5). Ordinary one-way ANOVA. (E) The ALP activity

and semi-quantitative estimation of calcium from Figure 4A were measured (n = 5). Ordinary one-way ANOVA. (F) OMSCs were co-transfected with shGATA4 and miR-206-3p mimic, the mRNA levels of *Opn*, *Ocn*, *Runx2*, *Alp* and *Osx* in the indicated cells were detected using qRT-PCR analysis (n = 5), and (G) ALP and ARS staining to detect osteogenic induction (n = 5). Ordinary one-way ANOVA. (H) miR-206-3p control inhibitor /inhibitor/ control mimic/ mimic transfected OMSCs were subjected to qRT-PCR analysis to detect the mRNA levels of Bmp3 (n = 5). Ordinary one-way ANOVA. (I) Protein and mRNA levels of Bmp3 from OMSCs of WT and cKO mice were examined by using western blotting and qRT-PCR analysis (n = 5). Two-tailed Student's t test. (J) OMSCs were co-transfected with miR-206-3p inhibitor and siBmp3, the mRNA levels of *Bmp3*, *Opn*, *Ocn*, *Runx2*, *Alp* and *Osx* in the indicated cells were detected using qRT-PCR analysis (n = 5). Ordinary one-way ANOVA. control inhibitor, Ctr-inhi; inhibitor, inhi; control mimic, Ctr-mimic. Each experiment was repeated at least three times with the same conditions. Data are shown as mean ± SD. $^{*}P < 0.05$, $^{**}P < 0.01$.

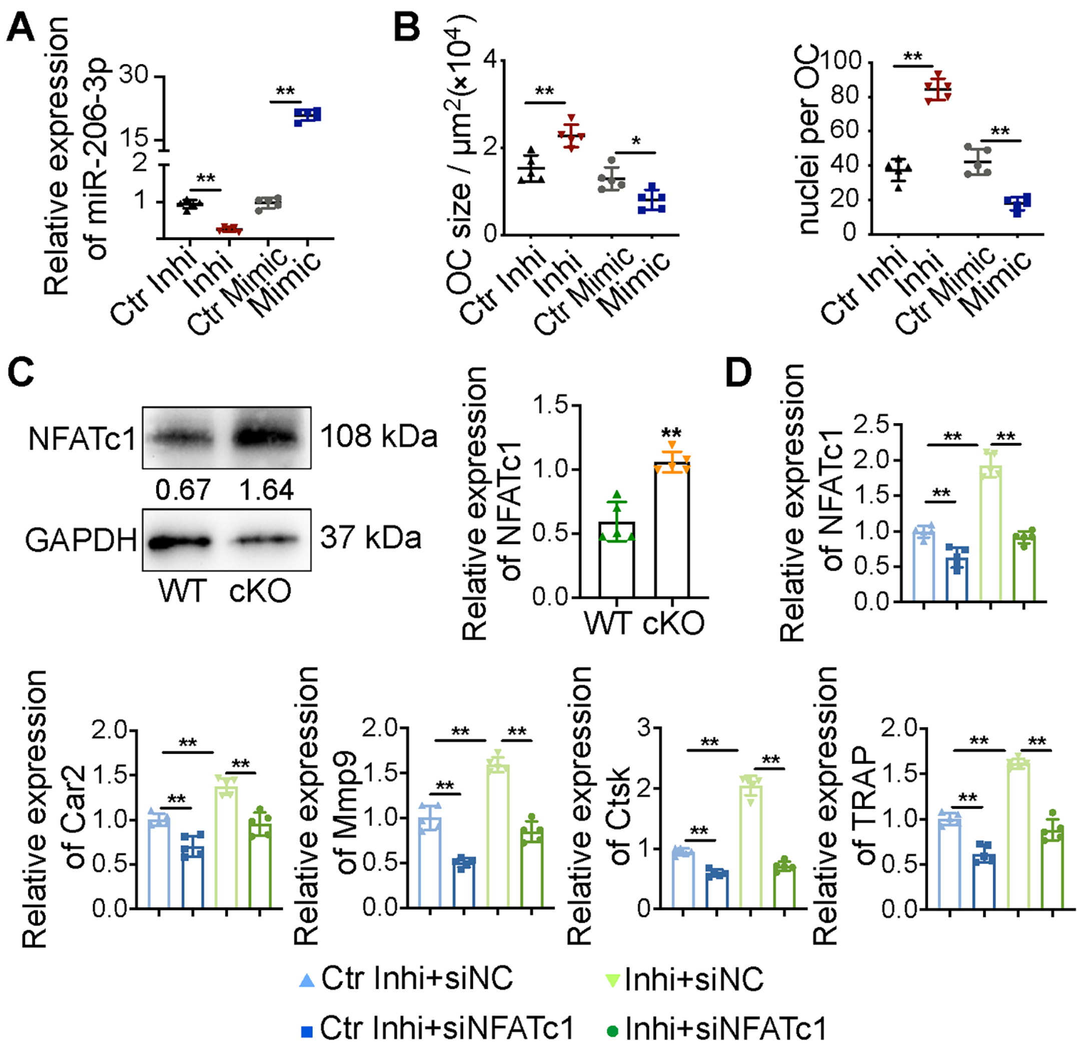

Figure S3. Exosomal miR-206-3p from OMSCs regulates osteoclast activity by targeting NFATc1.

(A) The exosomes from OMSCs were treated with miR-206-3p inhibitor or mimic, respectively, and then co-cultured with RANKL-treated RAW264.7 cells. The knockdown and over expression efficiency of miR-206-3p in osteoclasts was examined by qRT-PCR (n = 5). Ordinary one-way ANOVA. (B) The size and the nuclei numbers of TRAP-positive multinucleated cells ($TRAP^{+}$MNCs) in Figure 5A were measured (n = 5). Ordinary one-way ANOVA. (C) Protein and mRNA levels of NFATc1 of bone marrow-derived macrophages (BMMs) from *Gata4*$^{fl/fl}$ (WT) mice or

Wnt1-cre;Gata4$^{fl/fl}$ (cKO) mice were examined by using western blotting and qRT-PCR analysis (n = 5). Two-tailed Student's t test. (D) RANKL-treated RAW264.7 cells were co-transfected with miR-206-3p inhibitor and siNFATc1, mRNA levels of osteoclast related genes including NFATc1, Car2, Mmp9, Ctsk and TRAP were determined by using qRT-PCR assay. Ordinary one-way ANOVA. Each experiment was repeated at least three times with the same conditions. control inhibitor, Ctr-inhi; inhibitor, inhi; control mimic, Ctr-mimic. Data are shown as mean ± SD. $^{*}P < 0.05$, $^{**}P < 0.01$.

Table S1. Primers used

Gene	Primer	Sequence (5’-3’)
NFATc1	Forward<br>Reverse	CGAGTTCACATCCCACAG<br>GACAGCACCATCTTCTTCC
Car2	Forward<br>Reverse	ATCCTTGCTCCCTTCTTC<br>ATCCAGGTCACACATTCC
Mmp9	Forward<br>Reverse	TCACTTTCCCTTCACCTTC<br>ATTTGCCGTCCTTATCGT
Ctsk	Forward<br>Reverse	CCCATCTCTGTGTCCATC<br>AGTGCTTGCTTCCCTTCT
TRAP	Forward<br>Reverse	CAGCAGCCAAGGAGGACTAC<br>ACATAGCCCACACCGTTCTC
Opn	Forward<br>Reverse	ACCATGCAGAGAGCGAGGATT<br>GGGACATCGACTGTAGGGACG
Ocn	Forward<br>Reverse	ACTCTTGCCTCGTCCACT<br>GGTCTCTTCACTACCTCGCT
Runx2	Forward<br>Reverse	AGTTCCCAAGCTTTCATC<br>GGCAGGTAGGTGTGGTAGT
Alp	Forward<br>Reverse	CAGTGCGGTTCCAGACATAGT<br>GAACAGAACTGATGTGGAATACG
Osx	Forward<br>Reverse	CTACCCATCTGACTTTGCTC<br>CACTATTTCCCACTGCCTT
Rankl	Forward<br>Reverse	AACAGGCCTTTCAA GGAGCTGTGC<br>AAGAGGACAGACTCACTTTATGGGG
Bmp3	Forward<br>Reverse	AACGATGCTGCCATTTCT<br>CTTCCTCCTCTCAACCGA
GAPDH	Forward<br>Reverse	GAAGGTGAAGGTCGGAGTC<br>GAGATGGTGATGGGATTTC

Table S2. Differential genes

Upregulated miRNAs		Downregulated miRNAs
miR-6908-5p	miR-409-3p	miR-206-3p
miR-711	miR-6970-5p	miR-195a-3p
miR-6366	mir-5126	miR-466j
miR-2137	miR-6987-5p	miR-1187
miR-6349	miR-7081-5p	miR-466f
miR-7003-5p	miR-221-3p	miR-669e-5p
miR-99b-5p	miR-652-3p	miR-669f-5p
miR-8101	miR-1981-5p	miR-466m-5p
miR-145a-5p	miR-8119	miR-669m-5p
miR-674-5p	miR-714	miR-669n
miR-3102-5p.2-5p	miR-7653-5p	miR-669o-5p
miR-7654-3p	miR-5100	miR-665-5p
miR-7046-5p	miR-7684-5p	miR-466c-5p
miR-671-5p		miR-466f-5p
miR-425-5p		miR-16-1-3p
miR-5112		miR-1195
miR-30c-5p		miR-297a-5p
miR-3072-5p		miR-466h-5p
miR-7671-3p		miR-5129-3p
miR-3067-3p		miR-669a-5p
miR-34b-3p		miR-669p-5p
miR-574-3p		miR-669c-5p
miR-1249-5p		miR-574-5p
miR-151-3p		miR-3082-5p
miR-34c-3p		miR-669l-5p
miR-100-5p		mir-1194
miR-28a-3p		
miR-7687-5p		
miR-700-3p		
miR-6239		
miR-193b-3p		
miR-3473f		
miR-3104-5p		
miR-211-3p		
miR-423-5p		
miR-7221-3p		
miR-329-3p		
miR-6368		
miR-504-3p		
miR-222-3p		
miR-6910-5p		
